# A reduced M1-like/M2-like ratio of macrophages in healthy adipose tissue expansion during SGLT2 inhibition

**DOI:** 10.1038/s41598-018-34305-x

**Published:** 2018-10-31

**Authors:** Yasutaka Miyachi, Kyoichiro Tsuchiya, Kumiko Shiba, Kentaro Mori, Chikara Komiya, Naomi Ogasawara, Yoshihiro Ogawa

**Affiliations:** 1 Department of Molecular Endocrinology and Metabolism, Graduate School of Medical and Dental Sciences, Tokyo Medical and Dental University, Tokyo, Japan; 2 Third Department of Internal Medicine, Interdisciplinary Graduate School of Medicine and Engineering, University of Yamanashi, Kofu, Japan; 3 Department of Molecular and Cellular Metabolism, Graduate School of Medical and Dental Sciences, Tokyo Medical and Dental University, Tokyo, Japan; 4 Department of Medicine and Bioregulatory Science, Graduate School of Medical Sciences, Kyushu University, Fukuoka, Japan; 5 Japan Agency for Medical Research and Development, CREST, Tokyo, Japan

## Abstract

The adipose tissue includes various stromal cells, such as preadipocytes, endothelial cells, fibroblasts, and immune cells, which are involved in adipose tissue functions. We previously reported that, in obese mice, the sodium–glucose cotransporter 2 inhibitor ipragliflozin (Ipra) promoted the expansion of the epididymal adipose tissue (Epi) with increase of serum ketone body concentration. The Ipra-induced adipose tissue expansion did not deteriorate adipose inflammation, or systemic glucose/lipid metabolism, referred to as “healthy adipose tissue expansion.” Here we found that Ipra promoted healthy adipose tissue expansion with a reduced ratio of pro-inflammatory M1-like adipose tissue macrophages (ATMs) to anti-inflammatory M2-like ATMs. Ipra downregulated the gene expression of interleukin (IL)−15 (*Il15*) in stromal cells of Epi. IL-15 inhibited lipogenesis in 3T3-L1 cells associated with downregulation of the lipogenic gene. Ketone body β-hydroxybutyrate suppressed *Il15* gene induction in M1-polarized cultured macrophages, and a ketogenic diet reproduced the adipose tissue expansion without deteriorating systemic glucose metabolism in mice. Our data indicate that the phenotypic switch of ATMs could mediate healthy adipose tissue expansion by treatment with Ipra, and it may offer new insights into the pathophysiological mechanisms of adipose tissue expansion.

## Introduction

Obesity has become one of the major public health concerns of the past decades, being a key risk factor for type 2 diabetes, cardiovascular diseases, dyslipidemia, hypertension, and certain types of cancer, thereby leading to an increased mortality. Whereas the treatment for obesity and the prevention of obesity-related diseases are not always successful, a subgroup of obese individuals is at low risk for metabolic complications. “Metabolically healthy obesity (MHO)” represents such a subgroup of obese individuals who exhibit excessive accumulation of adipose tissue without adverse metabolic effects, including insulin resistance, glucose intolerance, and dyslipidemia^1^. MHO individuals are characterized by increased fat storage capacity of adipose tissue with anti-inflammatory phenotype, and decreased ectopic fat deposition in the liver and skeletal muscle; these morphological and functional changes in adipose tissue consequently inhibit the development of insulin resistance and cardiometabolic diseases.

Sodium–glucose cotransporter 2 (SGLT2) inhibitors are oral antidiabetic drugs that promote the urinary excretion of glucose by blocking its reabsorption in the renal proximal tubules. We previously reported that the SGLT2 inhibitor ipragliflozin (Ipra) promotes the expansion of epididymal adipose tissue (Epi) without deteriorating systemic glucose/lipid metabolism and adipose inflammation in obese mice^2,3^. This state of increased fat mass with a preserved metabolic fitness has been referred to as “healthy adipose tissue expansion”, which is similar to the adipose tissue found in MHO individuals.

A couple of studies with adipocyte-specific transgenic mouse models propose adipocyte-autonomous mechanisms to be responsible for healthy adipose tissue expansion; overexpression of adiponectin^4^ or the mitochondrial protein mitoNEET^5^, and ablation of phosphatase and tensin homolog (PTEN)^6^ exhibit severe adiposity without detrimental effects of high-fat diet (HFD) or *ob/ob* mutation on glucose and lipid metabolism. Besides mature adipocytes, the adipose tissue is composed of various stromal cells, such as preadipocytes, endothelial cells, fibroblasts, and immune cells, which change dramatically in number and cell type during the course of obesity^7,8^. Particularly among stromal cells, macrophage infiltration in obese adipose tissue is reported to precede the development of adipocyte hypertrophy^9^, suggesting that adipose tissue macrophages (ATMs) could regulate adipose expansion, inflammation, and systemic metabolism. ATMs are composed of at least two different phenotypes: classically activated M1-like macrophages and alternatively activated M2-like macrophages. M1-like ATMs produce proinflammatory cytokines, thus contributing to the induction of insulin resistance. In contrast, M2-like ATMs, which are the major phenotypes of ATMs in lean adipose tissue, mediate anti-inflammatory responses. Although these ATMs reportedly play several roles in the maintenance or improvement of systemic insulin sensitivity through inflammation of adipose tissue^10–12^, the stromal cell-mediated regulation of adipose expansion has not yet been fully understood.

In this study, we demonstrated that Ipra promoted the healthy adipose tissue expansion associated with a reduced M1-like/M2-like ratio of ATMs. Our observation implied that the change of M1-like/M2-like ratio of ATMs could cause adipocytes to induce healthy adipose tissue expansion during SGLT2 inhibition, and more broadly, it may offer new insights into the mechanisms of adipose expansion that could be therapeutic targets for obesity-associated metabolic comorbidities.

## Materials and Methods

### Animal experiments

Male wild-type (WT) C57BL/6 J mice were obtained from CLEA Japan. CCR2 knockout (KO) mice have been described previously^13^. Mice were maintained on a 12-h light–dark cycle. The animals were allowed free access to water and a standard diet (SD, CE-2; 343 kcal/100 g, 12.6% energy as fat; CLEA Japan, Inc.). Ipra (provided by Astellas Pharma Inc., Tokyo, Japan) was dissolved in dimethyl sulfoxide (DMSO; Nacalai Tesque, Inc., Kyoto, Japan) at 0.04% (v/v) and added into the drinking water. In the high-fat diet (HFD) feeding experiments, 6-week-old WT and CCR2 KO mice were fed a HFD (D12492; 524 kcal/100 g, 60% energy as fat; Research Diets, Inc., New Brunswick, NJ, USA) for 12 weeks, and were thereafter fed a HFD with the vehicle or Ipra for 4 weeks. In the SD feeding experiments, Ipra was administered to WT mice from 18- to 22-week-old. Calculated from water consumption, the average dose of Ipra was 7.6 mg/kg/day. GW2580 (LC Laboratories, Woburn, MA, USA) was dissolved at 34 mg/ml in 0.5% hydroxypropyl methylcellulose and 0.1% Tween 80. After 2 weeks of Ipra treatment, mice were given a daily oral administration of GW2580 (160 mg/kg) by a gavage for 2 weeks. Ketogenic (D12369B) and control diets (D12359) were purchased from Research Diets. Eight-week-old male WT mice were fed a ketogenic or control diet for 4 weeks. All animal experiments were approved by the Tokyo Medical and Dental University Committee on Animal Research (No. A2017-160A). All methods involving animals were performed in accordance with the relevant guidelines and regulations.

### Metabolic analysis

Blood glucose levels were measured using a glucometer (Glutest Pro R). Serum insulin and free fatty acid (FFA) concentrations were measured using enzyme-linked immunosorbent assay (Morinaga, Yokohama, Japan) and enzymatic method (Wako, Osaka, Japan), respectively. Beta hydroxybutyrate (BHB) levels were determined using a colorimetric method (ab83390, Abcam). Serum total cholesterol, triglyceride (TG), and alanine aminotransferase (ALT) concentrations were measured using Fuji Dry-chem 7000 V (Fujifilm Corporation, Tokyo, Japan). For glucose tolerance tests (GTT), mice were fasted for 16 h with free access to water, followed by intraperitoneal glucose injection (2 g/kg). We measured blood glucose concentrations at 0, 15, 30, 60, and 120 min after injection. Immeasurable high glucose concentration (>600 mg/dl) was recorded at 600 mg/dl. All analyses except for GTT were performed in the ad libitum fed state.

### Histology

Adipose tissue was fixed in 4% paraformaldehyde phosphate buffer solution for 24 h at room temperature. Deparaffinized sections (2 µm) were incubated with proteinase K for 5 min for antigen retrieval. Endogenous peroxidase activity was blocked with 0.3% H_2_O_2_ in methanol for 30 min. A rat anti F4/80 antibody (clone: A3-1, 1:1000 dilution: AbD Serotec) and a rabbit anti-polyclonal IL-15 antibody (1 µg/ml, PeproTech) was used to assess macrophage infiltration and to detect IL-15 expressing cells in the adipose tissue, respectively. The number of crown-like structures was counted in 15 different fields (×200) per slide and expressed as the mean number per field. IL-15 positive area was measured in 15–18 different fields (×200) per slide using Image J. Adipocyte size was quantified with Fiji (Image J) program Adiposoft 1.13 as previously described^3^.

### Cell culture

3T3-L1 cells were purchased from ATCC and maintained in Dulbecco modified Eagle medium (DMEM) containing 10% calf serum. Two days after reaching 100% confluency, cells were incubated in DMEM containing 10% fetal bovine serum (FBS), 5 µg/ml insulin, 0.5 mM isobutylmethyl xanthine, and 0.25 µM dexamethasone (day 0). After 2 days, the medium was replaced with DMEM containing 10% FBS and 5 µg/ml insulin (day 2). Thereafter, culture medium was replaced every 2 days with DMEM containing 10% FBS. The differentiated adipocytes were stimulated with recombinant murine IL-15 (50 or 250 ng/ml, Peprotech) at days 10, 12, and 14, as previously described^14^ with some modification. At day 15 of differentiation, cells were fixed with 4% paraformaldehyde phosphate buffer solution for 2 h at room temperature and washed with 60% isopropanol for 1 min. The fixed cells were stained with filtered Oil Red O solution for 2 h at room temperature. To quantify the lipid content, Oil Red O was eluted with 100% isopropanol and the absorbance of the extracts was measured at 540 nm using a microplate reader (Bio-Rad Laboratories, Hercules, CA, USA).

For isolation of peritoneal macrophages, 12-week-old male WT mice were intraperitoneally injected with 3% Brewer thioglycollate. Four days later, macrophages were collected by peritoneal lavage using cold DMEM containing 2% FBS. Cells were cultured for 3 h in DMEM containing 0.25% bovine serum albumin (BSA) and 1% penicillin/streptomycin. Cultures were washed twice with phosphate buffered saline (PBS) to remove nonadherent cells, pretreated with BHB (1 or 10 mM) in culture medium, and stimulated with lipopolysaccharide (LPS, 1 ng/ml), IL-4 (10 ng/ml, Biolegend), or IL-13 (10 ng/ml, Biolegend) overnight.

### Isolation of adipocytes and stromal vascular fraction cells

Epi was weighed, minced, and digested in 15 ml collagenase type 2 solution (2 mg/ml, Worthington) for 20 min at 37 °C with gentle shaking. The digestion mixture was centrifuged at 500 *g* for 3 min. Floating adipocytes were collected for ribonucleic acid (RNA) extraction and pellets containing stromal vascular fraction (SVF) cells were suspended in PBS. The suspension was passed through a 100-µm nylon mesh filter (BD Falcon) and centrifuged at 500 *g* for 3 min to pellet SVF cells.

### Isolation of peripheral blood leukocytes

Blood samples were obtained from the tail vein using capillary tubes. Blood was mixed with 0.5 M ethylenediaminetetraacetic acid (EDTA), lysed using ACK lysing buffer, and centrifuged at 500 *g* for 3 min.

### Flow cytometry and cell sorting

Isolated SVF cells or leukocytes were resuspended in 200 µl PBS containing 0.25% BSA, 0.2 mM EDTA, and 1% penicillin/streptomycin. Cells were preincubated for 7 min at 4 °C in Fc Block (CD16/32, BD Biosciences) and then stained for 15 min with fluorophore-conjugated antibodies at 4 °C. The following antibodies were used: anti-CD45 (clone: 30-F11, Biolegend), anti-F4/80 (clone: BM8, Biolegend), anti-CD11b (clone: M1/70, Biolegend), anti-CD11c (clone: N418, Biolegend), anti-CD206 (clone: C068C2, Biolegend), anti-CCR2 (clone: SA203G11, Biolegend), anti-Ly6G/Ly6C (clone: RB6-8C5, Biolegend), and anti-CD115 (clone: AFS98, Biolegend)^15^. Flow cytometric analysis was performed using a FACSCantoII (BD Biosciences). Cell sorting was performed with a FACSAriaII (BD Biosciences). Data were analyzed using FlowJo software (v9.4.10, Tree Star).

### Gene expression analysis

Total RNA was extracted using Qiazol (QIAGEN). cDNA synthesis and quantitative real-time polymerase chain reaction (PCR) were performed as described previously^16^.

### Microarray analysis

Total RNA extracted from Epi was purified using RNeasy MinElute Cleanup Kit (QIAGEN). Microarray analysis was performed at GeneticLab Co (Sapporo, Japan) using GeneChip Mouse Gene 2.0 ST Array (Affymetrix). The result was analyzed using Affymetrix Transcriptome Analysis ConsoleTM by GeneticLab Co. Gene ontology analysis was conducted using DAVID bioinformatics resources 6.8.

### Western blotting analysis

Epididymal adipose tissues were lysed by bead beating in a lysis buffer (2%SDS, 4 M Urea, 1 mM EDTA, 150 mM NaCl, 50 mM Tris pH 8.0), sonicated, and centrifuged. The lysates were separated by 8% SDS-PAGE and transferred to PVDF membranes. Western blotting was conducted using phospho (Ser473) – Akt antibody (9271, Cell Signaling Technology) and Akt antibody (9272, Cell Signaling Technology), followed by ECL detection (GE Lifescience). Band intensity was quantified using NIH Image J software.

### Statistical analysis

All data were analyzed using Graph Pad Prism 6 and were presented as mean ± standard error of mean. A p value < 0.05 was considered statistically significant. Unpaired *t*-test was used to compare two groups. One-way or two-way analysis of variance, followed by *post hoc* test, was used to compare more than two groups.

## Results

### Ipra induces healthy adipose tissue expansion with a reduced M1-like/M2-like ratio of ATMs in HFD-fed WT mice

**Figure 1:**
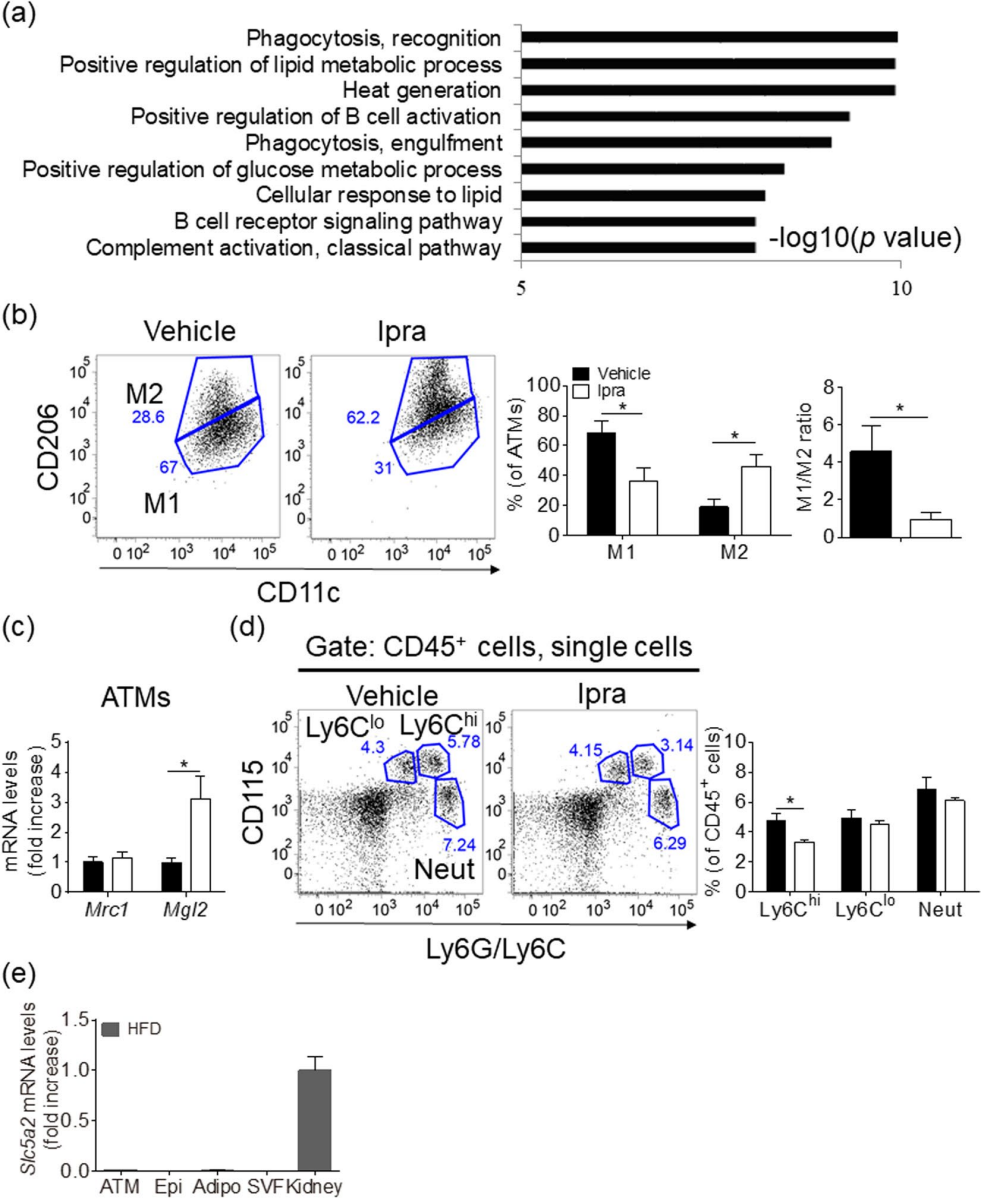
Characterization of ATMs in Ipra-treated WT mice fed a HFD. Six-week-old WT mice were fed a HFD for 16 weeks and were administered the vehicle (DMSO) or Ipra during the last 4 weeks. (**a**) The ontology terms enriched among the upregulated (>1.5-fold) mRNAs in Epi from HFD-fed mice treated with Ipra compared with those from HFD-fed mice treated with vehicle. The results are expressed as −log (p value). (**b**) Representative plots and quantification of flow cytometry for M1-like (CD11c^+^CD206^−^ cells) and M2-like (CD11c^−^CD206^+^ cells) adipose tissue macrophages (ATMs, CD45^+^CD11b^+^F4/80^+^ cells) in Epi, and M1-like/M2-like ratio (n = 4, each group). (**c**) Gene expression levels of CD206 (*Mrc1*) and CD301b (*Mgl2*) in the sorted ATMs (n = 4). (**d**) Representative plots and quantification of flow cytometry for Ly6C high (Ly6C^hi^) and low (Ly6C^l^°) monocytes, and neutrophils (Neut) in the peripheral blood (n = 4). (**e**) *Slc5a2* mRNA levels in sorted ATMs, Epi, isolated adipocytes (Adipo), stromal vascular fraction (SVF), and kidney of HFD-fed mice (n = 4). All values are presented as mean ± SEM. *p < 0.05 vs. vehicle.

As previously reported^2^, a four-week administration of Ipra significantly improved hyperglycemia and hyperinsulinemia without affecting the final body weight in HFD-induced obese mice (Supplementary Figure 1a). The liver weight was significantly decreased by Ipra compared with the vehicle, whereas Epi weight was increased^2^ (Supplementary Figure 1a). Ipra did not affect the weights of the inguinal and brown adipose tissue (data not shown). When WT mice were fed a SD, Ipra did not affect blood glucose, insulin, body weight, and weights of liver and Epi (Supplementary Figure 1b). Microarray analysis of Epi from HFD-fed mice revealed that Ipra upregulated genes related to phagocytosis, B-cell activation, and glucose and lipid metabolism (Fig. 1a). Flow cytometric analysis revealed that Ipra significantly decreased the proportion of M1-like ATMs (CD11c^+^CD206^−^ cells) and increased the proportion of M2-like ATMs (CD11c^−^CD206^+^ cells) in ATMs (CD45^+^CD11b^+^F4/80^+^ cells in SVF) (Fig. 1b and Supplementary Figure 1c), without alternation of the number of M1- and M2-like ATMs (Supplementary Figure 1d). Consequently, Ipra decreased the M1-like/M2-like ratio (Fig. 1b), accompanied by the upregulation of the M2-related gene *Mgl2* in ATMs (Fig. 1c). In addition, conditioned medium of adipose tissue from Ipra-treated HFD-fed mice increased *Mrc1* and *Mgl2* expression in peritoneal macrophages compared with vehicle-treated HFD-fed mice (Supplementary Figure 1e). The Ipra-induced reduction of the M1-like/M2-like ratio of ATMs was accompanied by the reduction of the population of Ly6C^hi^ monocytes (CD45^+^CD115^+^Ly6C^hi^ cells) in peripheral blood, which are preferentially recruited into inflamed tissues, where they are more likely to mature to M1-like macrophages^17^ (Fig. 1d). We assessed SGLT2 gene (*Slc5a2*) expression in the adipose tissue and in the cellular components of the adipose tissue to examine whether Ipra directly affects macrophage polarization through SGLT2. Expression levels of *Slc5a2* in ATMs, Epi, isolated adipocytes, and SVF cells were <1% as compared with those in the whole kidney (Fig. 1e).

### Pharmacological reduction of M2-like ATMs by blocking colony-stimulating factor-1 receptor signaling does not affect adipose tissue expansion in HFD-fed mice treated with Ipra

**Figure 2:**
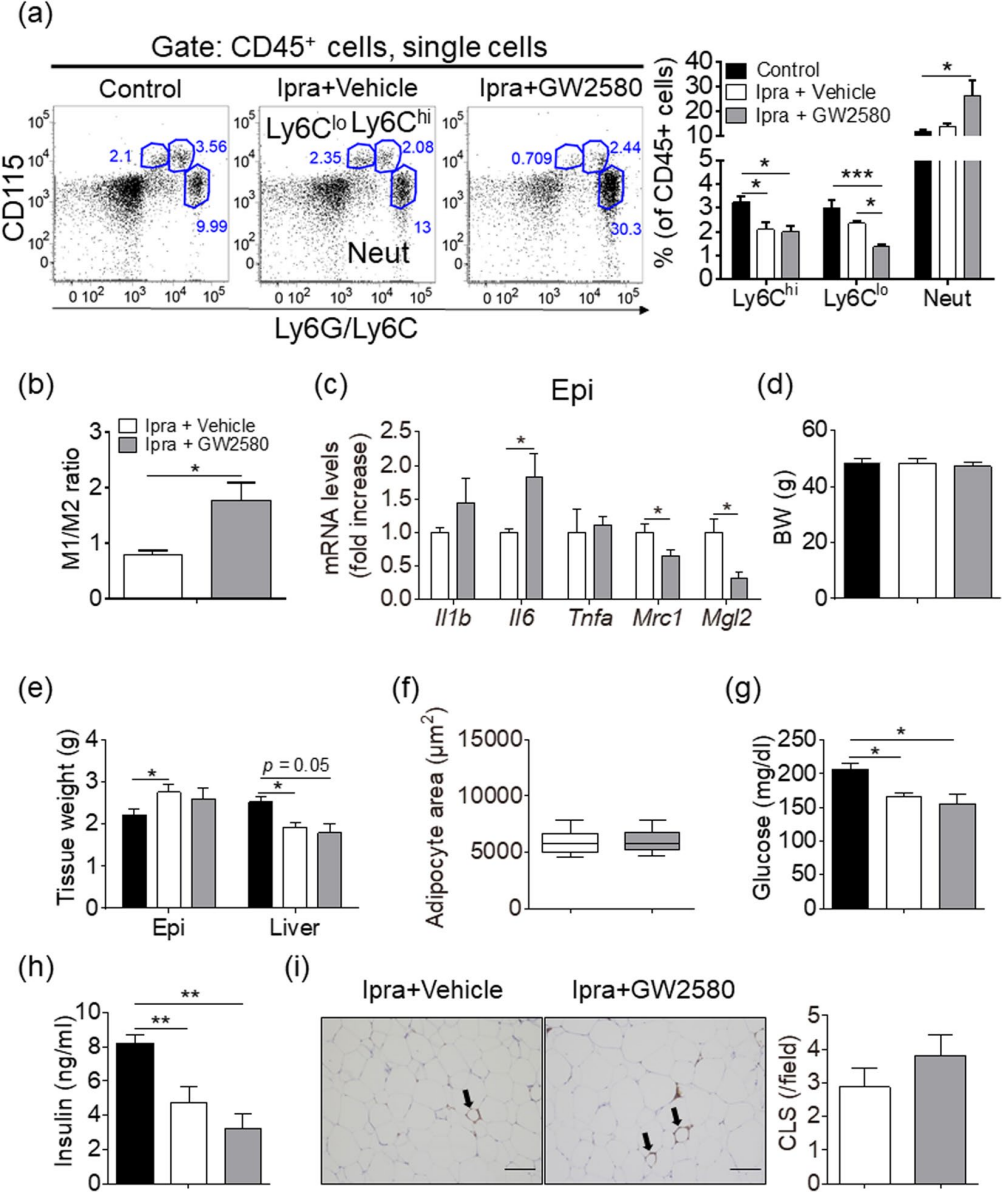
Metabolic phenotypes and characterization of ATMs in Ipra-treated WT mice fed a HFD treated with or without CSF-1 receptor blocker GW2580. Six-week-old WT mice were fed a HFD for 16 weeks and were given Ipra or DMSO (Control) during the last 4 weeks. CSF-1 receptor blocker GW2580 or vehicle were orally co-administered to Ipra-treated mice during the last 2 weeks. (**a**) Representative plots and quantification of flow cytometry for Ly6C^hi^ and Ly6C^l^° monocytes, and neutrophils (Neut) in the peripheral blood (n = 5, each group). (**b**) M1-like/M2-like ratio in Epi (n = 5). (**c**) Gene expression levels in Epi (n = 5). (**d**) Body weight, and (**e**) weights of Epi and liver (n = 5). (**f)** Adipocyte area in Epi (n = 5). (**g**) Blood glucose and (**h**) serum insulin levels in *ad libitum*-fed mice (n = 5). (**i**) Representative images of F4/80 immunostaining in Epi and the number of crown-like structure (CLS) (n = 5). Scale bar = 100 μm. All values represent mean ± SEM. *p < 0.05, **p < 0.01, ***p < 0.001 vs. indicated group.

We attempted to pharmacologically reduce M2-like ATMs by colony-stimulating factor 1 (CSF-1) receptor kinase inhibitor GW2580^18^ in order to examine whether the reduced M1-like/M2-like ratio of ATMs contributes to adipose tissue expansion in Ipra-treated mice fed a HFD. Administration of GW2580 to HFD-fed mice treated with Ipra reduced the percentage of Ly6C^lo^ without affecting that of Ly6C^hi^ in the peripheral blood monocytes (Fig. 2a). GW2580 significantly increased proportion of M1-like ATMs, and decreased the proportion and number of M2-like ATMs in Ipra-treated mice fed a HFD (Supplementary Figure 2a), resulting in an increase of M1-like/M2-like ratio of ATMs (Fig. 2b). The change associated with reduced expression of M2-related genes *Mrc1* and *Mgl2* in Epi (Fig. 2c). GW2580 downregulated these genes also in inguinal and brown adipose tissue (Supplementary Figure 2b), which is consistent with the significant reduction of the proportion of M2-like ATMs. Treatment with GW2580 did not affect the body weight, the weights of Epi and the liver, or adipocyte size in Epi of Ipra-treated mice fed a HFD (Fig. 2d–f). Blood glucose and serum insulin concentrations were also unaffected by GW2580 (Fig. 2g,h). GW2580 reduced serum FFA and TG concentrations at the fed state (Table 1). The number of crown-like structures (CLS) in Epi of Ipra-treated mice fed a HFD was negatively correlated with Epi weight (Supplementary Figure 2c), but treatment with GW2580 did not affect the number of CLS (Fig. 2i).

**Table 1:** Lipid profile of Ipra-treated HFD-fed mice after administration of GW2580.

	Ipra/vehicle	Ipra/GW2580
Serum FFA (mEq/l)	0.97 ± 0.07	0.70 ± 0.1*
Serum TG (mg/dl)	154.8 ± 5.6	134.4 ± 3.6*
Serum T-chol (mg/dl)	256.0 ± 14.3	255.2 ± 17.4

Ipra, ipragliflozin; FFA, free fatty acid; TG, triglyceride; T-chol, total cholesterol. Data are presented as mean ± SEM.

*p < 0.05 vs Ipra/vehicle. n = 5.

### CCR2 deficiency enhances Ipra-induced healthy adipose tissue expansion in HFD-fed mice

**Figure 3:**
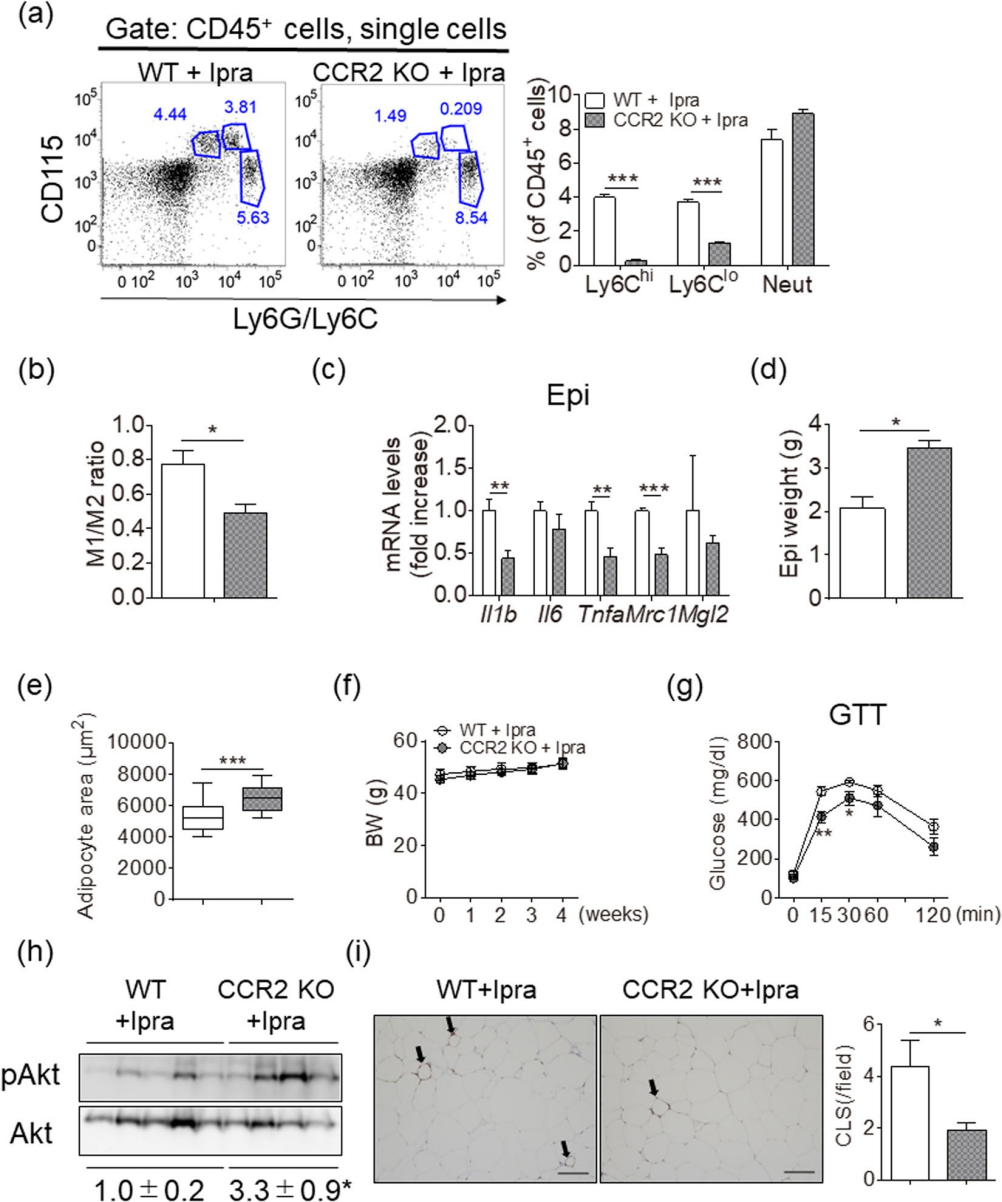
Metabolic phenotypes and characterization of ATMs in Ipra-treated mice fed a HFD with or without CCR2 KO background. Six-week-old WT and age-matched CCR2 KO mice were fed a HFD for 16 weeks and were given Ipra during the last 4 weeks. (**a**) Representative plots and quantification of flow cytometry for Ly6C^hi^ monocytes, Ly6C^l^° monocytes, and Neut in the peripheral blood of WT (n = 5) and CCR2 KO mice (n = 6) after 2 weeks of Ipra administration. (**b**) M1-like/M2-like ratio in Epi (n = 5–6). (**c**) Gene expression levels in Epi (n = 5–6). (**d**) Epi weight and (**e**) adipocyte area in Epi (n = 5–6). (**f**) BW changes in HFD-fed WT (n = 5) and CCR2 KO mice (n = 6) during Ipra treatment. (**g**) Intraperitoneal glucose tolerance test (GTT) of WT (n = 5) and CCR2 KO mice (n = 4) after 3 weeks of Ipra treatment. (**h**) Western blotting and quantification of phosphorylated Akt (pAkt) in Epi (n = 4–5). (**i**) Representative images and quantification of crown-like structures (CLS) in Epi of WT (n = 5) and CCR2 KO mice (n = 6). Scale bar = 100 µm. All values are presented as mean ± SEM. *p < 0.05, **p < 0.01, ***p < 0.001 vs. WT.

Subsequently, we assessed whether reduced proportion of M1-like ATMs could contribute to the healthy adipose tissue expansion of the Ipra-treated mice fed a HFD. We used CCR2 KO mice as a model of reduced circulating monocytes^19^ to decrease both M1- and M2-like ATMs in Ipra-treated mice fed a HFD; given that GW2580-induced reduction of the M2-related gene in Epi did not affect adipose tissue expansion in Ipra-treated mice, reduction of both M1- and M2-like ATMs was presumed to indirectly reveal the relevance of M1-like ATMs. Indeed, flow cytometric analysis of peripheral blood after 2 weeks of SGLT2 inhibition confirmed the significant reduction of both Ly6C^hi^ and Ly6C^lo^ monocytes in HFD-fed CCR2 KO mice (Fig. 3a). CCR2 deficiency markedly decreased the total number of ATMs, and M1- and M2-like ATMs normalized based on Epi weight (Supplementary Figure 3a). CCR2 deficiency significantly decreased the proportion of M1-like ATMs, and increased the proportion of M2-like ATMs in Ipra-treated mice fed a HFD (Supplementary Figure 3b), resulting in an decrease of M1-like/M2-like ratio of ATMs (Fig. 3b). Ipra treatment itself had little effect on CCR2 expression in circulating monocytes of HFD-fed mice (Supplementary Fig. 3c). Both the M1- (*Il1b* and *Tnfa*) and M2-related genes *Mrc1* were suppressed in Epi of CCR2 KO mice as compared with WT mice fed a HFD (Fig. 3c). CCR2 deficiency significantly increased Epi weight with increased adipocyte size in Ipra-treated mice fed a HFD (Fig. 3d,e). CCR2 KO mice exhibited a trend of decrease in liver weight with a trend of decrease in serum ALT concentration (Supplemental Fig. 3d and e), which resulted in comparable body weight between CCR2 KO and WT mice fed a HFD under Ipra treatment (Fig. 3f). Despite increased adipose tissue mass, CCR2 deficiency improved glucose tolerance (Fig. 3g), and even elevated Akt phosphorylation and reduced CLS formation in Epi (Fig. 3h,i, Supplementary Figure 4). CCR2 deficiency reduced serum cholesterol concentrations, whereas it did not change serum FFA and TG concentrations (Table 2).

**Table 2:** Lipid profile of Ipra-treated HFD-fed WT and CCR2 KO mice.

	WT/Ipra	CCR2 KO/Ipra
Serum FFA (mEq/l)	1.12 ± 0.08	1.05 ± 0.05
Serum TG (mg/dl)	173.2 ± 13.5	215.5 ± 23.3
Serum T-chol (mg/dl)	250.4 ± 5.3	212.3 ± 15.2*

Ipra, ipragliflozin; FFA, free fatty acid; TG, triglyceride; T-chol, total cholesterol. Data are presented as mean ± SEM.

*p < 0.05 vs WT/Ipra. n = 5–6.

### Ketone body attenuates *Il15* upregulation in M1-polarized macrophages

**Figure 4:**
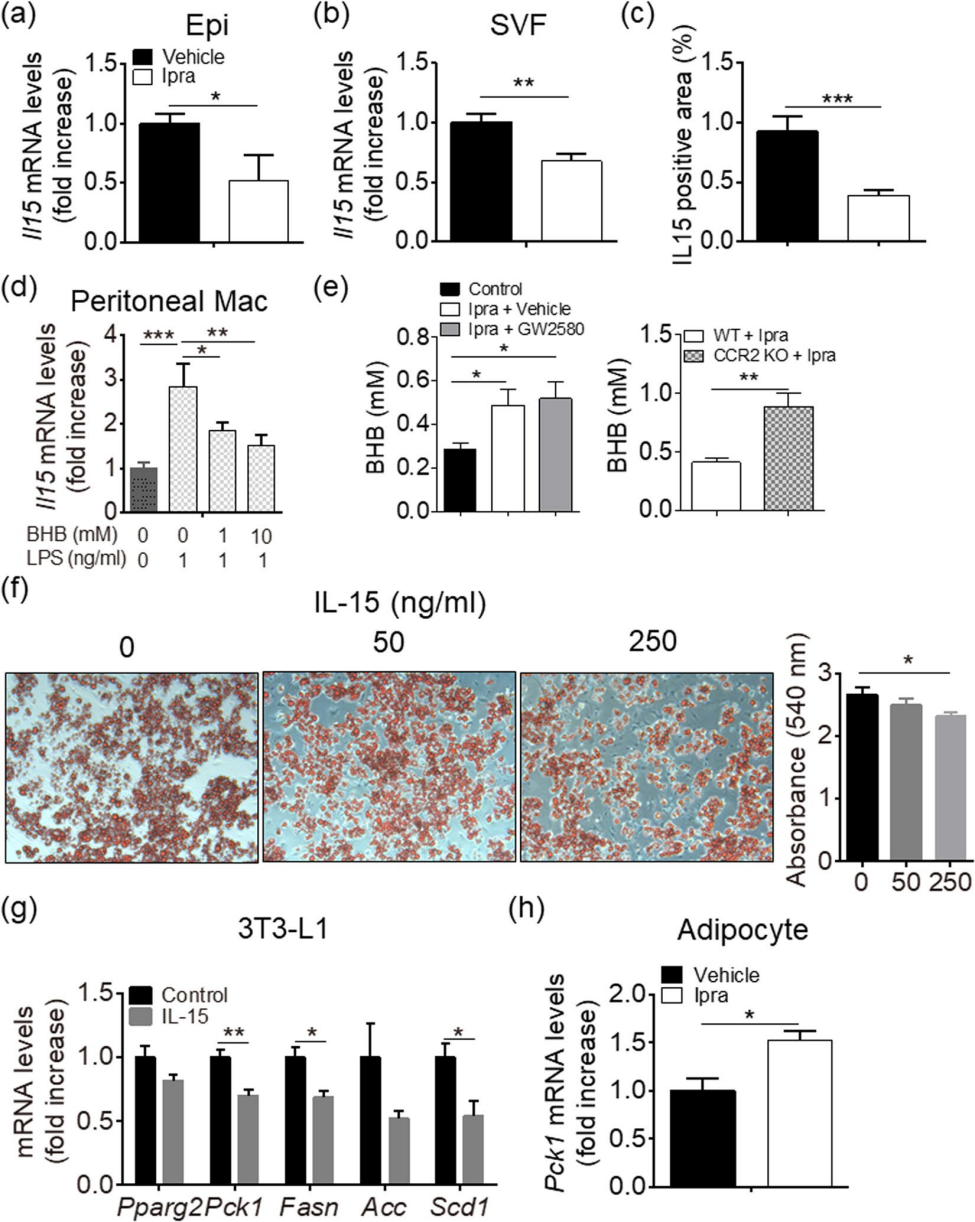
Effects of IL-15 on lipid accumulation in 3T3-L1 cells. IL-15 (*Il15*) mRNA in the (**a**) Epi and (**b**) SVF from Epi of HFD-fed mice after 4 weeks of Ipra or vehicle treatment (n = 5). (**c**) IL-15 positive area in Epi of HFD-fed WT mice after 4 weeks of Ipra or vehicle treatment (n = 5). (**d**) *Il15* mRNA levels in the peritoneal macrophages stimulated by lipopolysaccharide (LPS, 1 ng/ml) with or without pretreatment with beta-hydroxybutyrate (BHB, 1 or 10 mM) (n = 4). (**e**) Serum BHB levels in GW2580-treated mice (n = 5) and CCR2 KO mice (n = 6) fed a HFD under Ipra treatment. (**f**) 3T3-L1 cells were stimulated by recombinant murine IL-15 (50 or 250 ng/ml) at days 10, 12, and 14 of differentiation. Representative images and quantification of Oil Red O staining of 3T3-L1 at day 15 (n = 4). (**g**) mRNA levels of *Pparg2*, *Pck1*, *Fasn*, *Acc*, and *Scd1* in 3T3-L1 cells stimulated by IL-15 (250 ng/ml) for 24 h at day 14 (n = 4). (**h**) *Pck1* mRNA levels in the isolated adipocytes from Epi of HFD-fed mice treated with Ipra or vehicle (n = 4). All values are presented as mean ± SEM. *p < 0.05, **p < 0.01, ***p < 0.001 vs. indicated group.

We explored the molecular mechanism of ATM-mediated healthy adipose tissue expansion during SGLT2 inhibition, in terms of the functional interaction between ATMs and adipocytes. Based on microarray data in Epi from Ipra-treated mice (Supplementary Table 1), we focused on IL-15 (*Il15*) as a potential negative regulator of healthy adipose tissue expansion. Quantitative real-time RT-PCR analysis confirmed that Ipra significantly decreased *Il15* expression in Epi of HFD-fed mice (Fig. 4a). To narrow down the cellular source of IL-15 in adipose tissue, we separated Epi into adipocytes and SVF. Although Ipra did not affect *Il15* expression in the adipocyte fraction (Supplementary Figure 5a), it significantly decreased *Il15* expression in SVF (Fig. 4b). Furthermore, immunohistochemical analysis revealed that IL-15-positive cells were mostly found around adipocytes in HFD-fed mice (Supplementary Figure 5b), and that the number of them was significantly reduced by Ipra treatment (Fig. 4c). The localization of IL-15-positive cells appears to be similar to CLS, suggesting that the main cellular source of IL-15 in adipose tissue can be macrophages. In primary cultures of peritoneal macrophages from WT mice, LPS-induced M1 polarization significantly induced *Il15* expression along with other inflammatory genes (Fig. 4d and Supplementary Figure 5c). In contrast, IL-4- or IL-13-induced M2 polarization significantly inhibited *Il15* expression (Supplementary Figure 5d).

With regard to a possible factor that inhibits *Il15* expression in SVF cells during Ipra treatment, we focused on ketone body BHB, whose serum concentration is increased in Ipra-treated mice^2^. BHB significantly attenuated *Il15* induction in M1-polarized peritoneal macrophages (Fig. 4d), without affecting *Il1b*, *Il6*, or *Tnfa* induction in M1-polarized peritoneal macrophages (Supplementary Figure 5c). In HFD mice treated with Ipra, whereas GW2580 treatment did not change serum BHB concentration, CCR2 KO mice showed increase of serum BHB concentrations (Fig. 4e).

### IL-15 inhibits lipid accumulation in 3T3-L1 cells with *Pck1* downregulation

Consistent with a previous report^14^, recombinant IL-15 dose-dependently inhibited lipid accumulation in 3T3-L1 adipocytes (Fig. 4f), in association with a reduced expression of lipogenic genes, such as *Pck1*, *Fasn*, and *Scd1* (Fig. 4g). Among these lipogenic genes, *Pck1*, which encodes phosphoenolpyruvate carboxykinase (PEPCK), has been reported to mediate glyceroneogenesis in adipocytes, thus promoting TG accumulation by fatty acid re-esterification. Increased fatty acid re-esterification by PEPCK overexpression in adipose tissue has been reported to lead to adipose expansion without insulin resistance^20^, suggesting that PEPCK-dependent TG accumulation could contribute to healthy adipose expansion. In adipocytes from Epi of Ipra-treated mice, *Pck1* expression was significantly increased relative to vehicle-treated mice (Fig. 4h). A similar result was obtained in Epi from the CCR2 KO mice treated by Ipra (Supplementary Figure 5e). In contrast, *Pck1* expression was decreased in Epi of HFD-induced obese mice compared with SD-fed mice (Supplementary Figure 5f).

### Ipra alters ceramide and sphingomyelin subspecies in adipose tissue of HFD-fed mice

We performed lipidomics profiling of Epi to assess whether the healthy adipose expansion accompanied altered lipid profiles including individual lipid classes. Ipra treatment decreased ceramide (CER) [FA24:1] and sphingomyelin (SM) [FA24:1], and increased SM [FA18:0] in HFD-fed mice (Supplementary Figure 6a). CCR2 deficiency in HFD-fed mice during Ipra treatment also had similar effects on CER and SM subspecies in Epi (Supplementary Figure 6b). Ipra had little effect on di- and triacylglycerol, or free-fatty acid subspecies in Epi of HFD-fed mice (data not shown).

### Ketogenic diet phenocopies adipose tissue expansion in Ipra-treated mice

**Figure 5:**
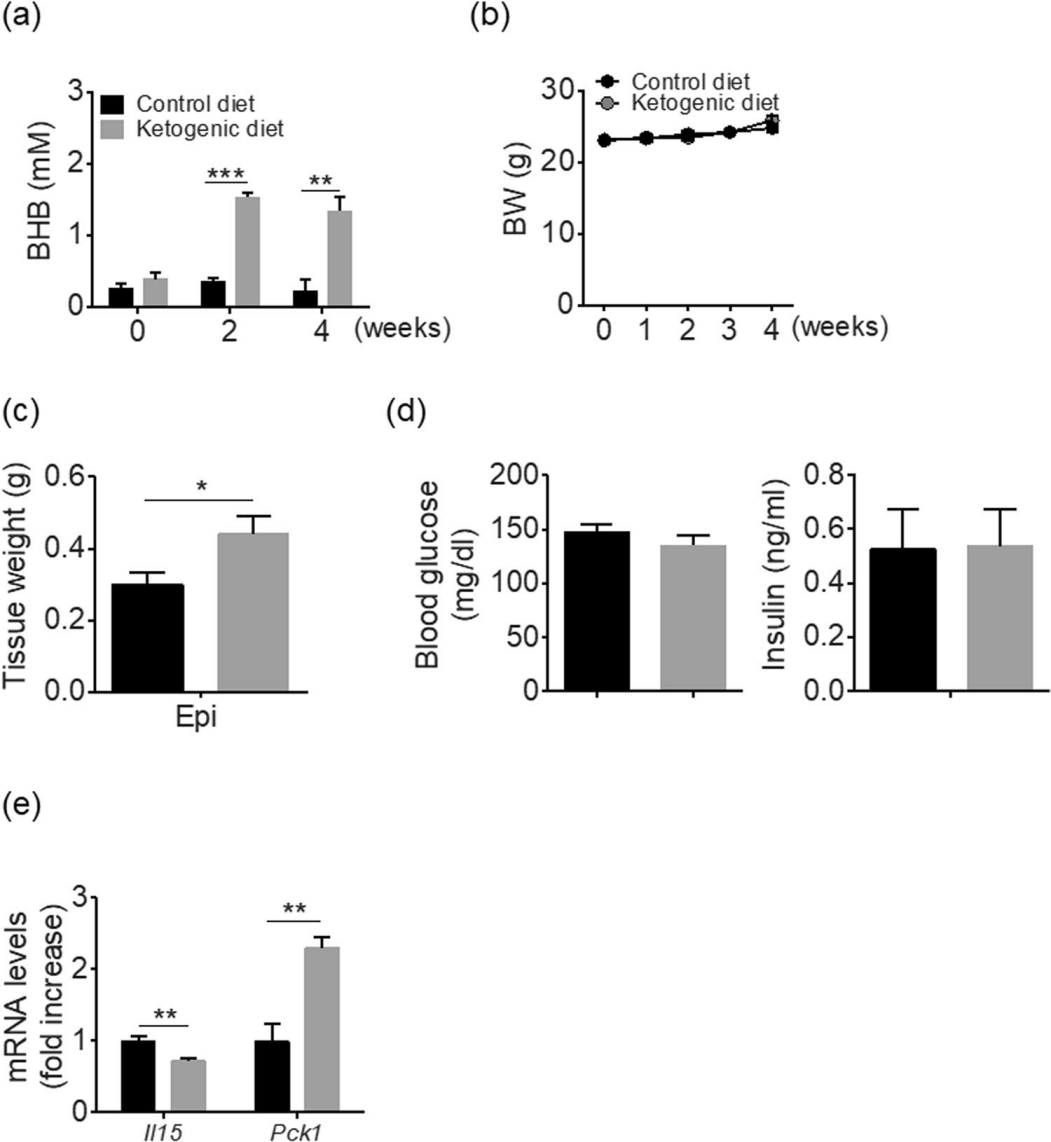
Metabolic phenotypes of ketogenic diet-fed WT mice. Eight-week-old WT mice were fed a ketogenic or control diet for 4 weeks. (**a**) Serum BHB levels and (**b**) changes in BW (n = 5). (**c**) Epi weight. (**d**) Blood glucose and serum insulin levels in ad libitum-fed mice. (**e**) mRNA levels of *Il15* and *Pck1* in Epi. All values are presented as mean ± SEM. *p < 0.05, **p < 0.01, ***p < 0.001 vs. control diet.

We finally examined whether ketone bodies could cause adipose expansion without deteriorating systemic glucose metabolism *in vivo*, and whether it accompanies a decreased *Il15* as observed in cultured M1-polarized peritoneal macrophages (Fig. 4d). Eight-week-old male WT mice were fed a ketogenic diet for 4 weeks; after 2 weeks of the ketogenic diet, serum BHB levels were significantly increased as compared with the control diet (Fig. 5a). The ketogenic diet did not change the body weight in WT mice as compared to those fed the control diet (Fig. 5b). The ketogenic diet significantly increased Epi weight (Fig. 5c), without inducing hyperglycemia or hyperinsulinemia (Fig. 5d). Furthermore, the ketogenic diet significantly suppressed *Il15* expression with an increased *Pck1* expression in Epi (Fig. 5e). Lipidomics analysis confirmed, similar to Ipra and CCR2 deficiency, the ketogenic diet decreased CER [FA24:1] and SM [FA24:1], and increased SM [FA18:0] in Epi (Supplementary Figure 6c).

## Discussion

The healthy adipose tissue expansion of Ipra-treated HFD-fed mice reported in our previous^2^ and the present studies are partly explained by a cell-autonomous mechanism demonstrated in a previous report; adipocyte-specific PTEN-knockout mice, which exhibit enhanced insulin signaling in adipocytes, gained more weight and adiposity during HFD feeding^6^. The mice showed enhanced insulin sensitivity, improved hepatic steatosis, and reduced adipose tissue inflammation, which were consistent with this study. Therefore, we consider that the enhanced adipocyte insulin signaling by Ipra, probably via improving hyperinsulinemia, results in increased lipid-storage capacity in adipocytes. Since it has been reported that hyperglycemia also causes insulin resistance and inflammation in adipocyte with oxidative stress-dependent manner^21^, reducing hyperglycemia by Ipra could additionally contribute to improve insulin resistance and inflammation in adipocytes. In addition to such an adipocyte-autonomous mechanism, the data of this study indicate a novel ATM-mediated mechanism for healthy adipose tissue expansion during SGLT2 inhibition, and this study may also reveal the role of ATMs in the regulation of energy storage through fat accumulation. Clinically, the involvement of ATMs in the development of adipose tissue may be possibly observed in MHO subjects. Similarly, their potential as a therapeutic target of obesity-associated metabolic comorbidities through the induction of healthy adipose tissue expansion can be explored.

Considered from previous papers reporting the effects of SGLT2 inhibitors on HFD-fed mice, the effects of SGLT2 inhibitors on body weight and adiposity vary across the studies; treatment with tofogliflozin suppressed HFD-induced body weight gain as well as the progression of hepatic steatosis, when it is administered for 20 weeks beginning at the start of HFD feeding^22^. In contrast, 4-week treatment with remogliflozin following 11-week of HFD feeding attenuated hepatic steatosis without changing body weight gain^23^. Other report has shown that luseogliflozin decreased liver weight and serum ALT levels in STZ-treated mice fed a HFD, without affecting body weight gain^24^. Taken together, although the precise mechanisms remain unknown, these observations suggest that the study protocol, especially in the timing of administration of SGLT2 inhibitors during HFD feeding and a mouse model, could affect body weight gain in mice.

As well as rodent, a human study has demonstrated that the SGLT2 inhibitor empagliflozin elicited an adaptive increase of energy intake in patients with type 2 diabetes, which caused substantially less weight loss than that expected from the energy dissipated because of glycosuria^25^. It suggests that inhibition of SGLT2 basically causes similar biological responses of appetite and energy intake in human and mice. Although previous human studies^26–28^ have consistently shown that treatment of SGLT2 inhibitors reduces body weight as average, our previous data has demonstrated that approximately 20% of patients treated with Ipra did not achieve more than 1% of body weight reduction^2^. While studies in humans predominantly describe weight loss with SGLT2 inhibitor treatment, the effect of SGLT2 inhibitors on body weight and adiposity in murine studies has been variable.

Other anti-diabetic agents, such as thiazolidinedione^10^, dipeptidyl peptidase-4 inhibitor^29^, and metformin^30^ have been reported to have direct effects on ATMs by the induction of an M2-dominant shift. However, considering the low expression levels of *Slc5a2* in macrophages, adipocytes, SVF cells and adipose tissue, Ipra is unlikely to have the direct effects on such cells or on a tissue. In the context of obesity-associated metabolic comorbidities, hyperglycemia has been shown to interfere with IL-4 action to M2-like polarization in macrophages as assessed by the decreased expression of M2-related genes and the reduced functional activity of arginase^31^. Therefore, the improvement of hyperglycemia could be considered as one of the mechanisms for changing M1-like/M2-like ratio of ATMs in Ipra-treated mice.

The pharmacological blockade of CSF-1 receptor signaling by GW2580 decreased M2-related genes in Epi of Ipra-treated mice; however, it did not affect adipose mass or glucose metabolism. In contrast, decrease of both M1- and M2-like ATMs in Epi of Ipra-treated CCR2 KO mice more strongly enhanced adipose tissue expansion without deteriorating glucose intolerance, than those of only Ipra-treated WT mice. Some previous reports have shown that M2-like macrophages promote adipose tissue expansion in mice; deficiency of Trib1, an adaptor protein involved in protein degradation by interacting with an ubiquitin ligase, diminishes adipose tissue mass accompanied with a reduction of M2-like ATMs^32^. A recent report has shown that CD206-positive cells are primarily M2-like macrophages, and that ablation of CD206-positive M2-like macrophages improves systemic insulin sensitivity with an increased number of smaller adipocytes^33^. Although the exact reason of the discrepancies of phenotypes between these previous studies and ours remains unclear, we assume that it might be due to the methodology for reduction of M2-like ATMs. In the present study, we used CSF-1 receptor kinase inhibitor GW2580 to reduce M2-like ATMs. Although the phenotypes of GW2580-treated mice were inconsistent with those of Trib1-defecient and CD206-positive cells-depleted mice, they were consistent with phenotypes of *op*/+ mice, which carry an inactivating heterozygous mutation in the CSF-1 gene^34^; HFD-fed *op*/+ mice showed no change in adiposity, glucose tolerance, or adipose inflammation compared to HFD-fed wild-type mice. Although these genetic or pharmacological approaches successfully reduced M2-like ATMs assessed as representative marker genes and/or surface antigens, the reduced cell population might not be identical, possibly leading to the differential effects on adiposity.

Among M1-related genes in adipose tissue altered by Ipra treatment, *Il15* is significantly downregulated in SVF of Ipra-treated mice. Consistent with a previous report^35^, immunostaining for IL-15 in Epi suggests that ATMs are assumed to be the cellular source of IL-15 in SVF. *Il15* ablation in mice has been reported to result in a significant increased weight gain independent of appetite; notably, the mice do not display obesity-associated inflammation, characterized as an increase of serum IL-6 and tumor necrosis factor-α concentrations^36^. In addition, consistent with our observation, treatment of differentiated human adipocytes with recombinant IL-15 resulted in decreased lipid deposition, indicating its direct inhibitory effect on adipogenesis^14^. Although the pathological relevance of IL-15 in the healthy adipose tissue expansion requires further studies, the Ipra-induced downregulation of *Il15* in SVF cells, possibly in ATMs, seems to promote adipocyte expansion in a paracrine manner.

In addition to the reduced M1-like/M2-like ratio of ATMs, we consider that ketone bodies contribute to *Il15* downregulation in the SVF cells of adipose tissue from Ipra-treated mice. We previously showed that 4 weeks of Ipra treatment significantly increased serum BHB concentrations in HFD-fed mice^2^. As well as the HFD-fed WT mice^2^, BHB concentrations and the adipose tissue expansion during SGLT2 inhibition were changed in parallel also in GW2580-treated and CCR2 KO mice; both BHB concentration and adipocyte size were increased in CCR2 KO mice, but neither of them were increased in GW2580-treated mice. BHB has been shown to increase global histone acetylation by inhibiting class I histone deacetylases (HDAC) in mouse tissues^37^. Inhibition of HDAC by BHB was correlated with global changes in transcription of the genes encoding oxidative stress resistance factors; BHB treatment consequently protected mice from oxidative stress. Furthermore, BHB is also demonstrated to abolish inflammatory responses in human macrophages by inhibiting NLRP3 inflammasome^38^. Taken together, it is conceivable that the BHB-induced inhibition of *Il15* in M1 macrophages may be mediated at least partly by attenuation of oxidative stress and/or NLRP3 inflammasome. Given that BHB did not suppress the elevation of *Tnfa* and *Il6* in M1-skewed macrophages, the inhibitory effect of BHB on the induction of M1-related genes seems to be gene-selective. The precise molecular mechanisms by which BHB modulates inflammatory responses in macrophages need further studies.

Since adipose tissue conditioned medium from Ipra-treated HFD-fed mice increased *Mrc1* and *Mgl2* expression in peritoneal macrophages compared with vehicle-treated HFD-fed mice, adipose tissue-derived substances, as well as ketone body, are considered as possible distinct and unique factors to induce Ipra-induced functional modulation of ATMs. Various signaling molecules released from dying cells are known to act as chemoattractants and affect immune responses^39–41^. In adipose tissues, we previously showed that macrophage-inducible C-type lectin (Mincle), a pathogen sensor for *Mycobacterium tuberculosis*, is localized to ATMs constituting CLS and activated by an endogenous ligand released from dying adipocytes^42^. Mincle activation induces inflammatory responses and extracellular matrix production in macrophages, which may deteriorate adipose tissue inflammation and limit HFD-induced hypertrophy of adipocytes to inhibit ectopic lipid accumulation. Therefore, substances from dying and/or dead adipocytes have been also proposed to locally affect ATMs’ characters, and reduced adipocyte death induced by Ipra may affect the phenotypes of ATMs.

Because *Pck1* upregulation in adipose tissue and adipocytes is commonly observed in HFD-fed WT mice treated with Ipra, CCR2 KO mice and ketogenic diet-fed mice, adipose *Pck1* may play a key role in the induction of healthy adipose expansion. In addition, IL-15 inhibited lipid accumulation in 3T3-L1 cells with downregulation of *Pck1*, suggesting IL-15 to be an exogenous factor, possibly produced from ATMs *in vivo*, to suppress lipogenesis. Adipocyte PEPCK is known to mediate glyceroneogenesis, which synthesizes glycerol-3-phosphate or TG from precursors other than glucose. Some reports proposed that PEPCK-mediated glyceroneogenesis plays a critical role in the development of healthy adipose tissue expansion; *aP2*-driven PEPCK overexpression increases adipose mass with enhanced fatty acid re-esterification and without worsening systemic glucose metabolism^20^. Mice carrying the mutated binding site of PPARγ in the promoter region of a cytosolic PEPCK (*PEPCK*-*C*) gene, which consequently abolishes *PEPCK-C* gene expression in adipose tissue, exhibit reduced TG deposition in Epi^43^. Considering that adipose tissue expansion by HFD feeding conversely suppressed *Pck1* expression, adipose *Pck1* and PEPCK-mediated glyceroneogenesis may be a possible determinant factor in healthy adipose tissue expansion. The pathophysiological significance and detailed molecular mechanisms of PEPCK-mediated glyceroneogenesis in the healthy adipose tissue expansion and the involvement of the ATMs-derived IL-15 in adipocyte *Pck1* regulation require further studies.

Along with the possible alternation of the lipid synthesis pathway toward PEPCK-mediated glyceroneogenesis, Ipra treatment also affected profiles of CER and SM subspecies in Epi of HFD-fed mice. CER and SM, which are included as part of sphingolipids, have been demonstrated to play a substantial role in the development of a variety of diseases as important bioactive mediators. In adipose tissue, adipocyte-specific ablation of SPTLC2, a subunit of serine palmitoyltransferase that is an initial enzyme in sphingolipid biosynthesis, has been reported to increase CER[C24:1] content in adipose tissue of HFD-fed mice, and reduce adipose tissue mass with marked *Pck1* downregulation^44^. In addition, the adipocyte-specific SPTLC2-knockout mice also accompanied hepatic steatosis, implying a mirror image phenotype of lipid deposition and content as the Ipra-treated mice in our study. Since CCR2 deficiency in HFD-fed mice during Ipra treatment and ketogenic diet also increased CER[C24:1] in Epi, the healthy adipose expansion, at least observed in these conditions, could be mediated by alternation of the CER subspecies. Although the link between PEPCK-mediated glyceroneogenesis and sphingolipid biosynthesis, and precise role of sphingolipid in the healthy adipose expansion remain unclear, alternation of lipid synthesis and subspecies may be involved in the development of the healthy adipose expansion.

In conclusion, this study revealed the ATM-mediated mechanisms by which SGLT2 inhibition promoted healthy adipose expansion. Furthermore, ATMs are proposed to be potential therapeutic targets for obesity-associated metabolic comorbidities via the changes they induce in the characteristics of adipose tissue.

## Electronic supplementary material

Supplementary Information

## References

[1] Stefan Norbert, Häring Hans-Ulrich, Schulze Matthias B (2018). Metabolically healthy obesity: the low-hanging fruit in obesity treatment?. The Lancet Diabetes & Endocrinology.

[2] Komiya C (2016). Ipragliflozin Improves Hepatic Steatosis in Obese Mice and Liver Dysfunction in Type 2 Diabetic Patients Irrespective of Body Weight Reduction. PLoS One.

[3] Shiba K (2018). Canagliflozin, an SGLT2 inhibitor, attenuates the development of hepatocellular carcinoma in a mouse model of human NASH. Scientific reports.

[4] Kim JY (2007). Obesity-associated improvements in metabolic profile through expansion of adipose tissue. J Clin Invest.

[5] Kusminski CM (2012). MitoNEET-driven alterations in adipocyte mitochondrial activity reveal a crucial adaptive process that preserves insulin sensitivity in obesity. Nat Med.

[6] Morley TS, Xia JY, Scherer PE (2015). Selective enhancement of insulin sensitivity in the mature adipocyte is sufficient for systemic metabolic improvements. Nature communications.

[7] Suganami T, Ogawa Y (2010). Adipose tissue macrophages: their role in adipose tissue remodeling. J Leukoc Biol.

[8] Suganami T, Nishida J, Ogawa Y (2005). A paracrine loop between adipocytes and macrophages aggravates inflammatory changes: role of free fatty acids and tumor necrosis factor alpha. Arterioscler Thromb Vasc Biol.

[9] Sekimoto R (2015). Visualized macrophage dynamics and significance of S100A8 in obese fat. Proc Natl Acad Sci USA.

[10] Fujisaka S (2009). Regulatory mechanisms for adipose tissue M1 and M2 macrophages in diet-induced obese mice. Diabetes.

[11] Fujisaka S (2011). Telmisartan improves insulin resistance and modulates adipose tissue macrophage polarization in high-fat-fed mice. Endocrinology.

[12] Lackey DE, Olefsky JM (2016). Regulation of metabolism by the innate immune system. Nature reviews. Endocrinology.

[13] Ito A (2007). Role of MAPK phosphatase-1 in the induction of monocyte chemoattractant protein-1 during the course of adipocyte hypertrophy. J Biol Chem.

[14] Barra NG (2014). Interleukin-15 modulates adipose tissue by altering mitochondrial mass and activity. PLoS One.

[15] Tsuchiya K (2013). Expanded granulocyte/monocyte compartment in myeloid-specific triple FoxO knockout increases oxidative stress and accelerates atherosclerosis in mice. Circ Res.

[16] Miyachi Y (2017). Roles for Cell-Cell Adhesion and Contact in Obesity-Induced Hepatic Myeloid Cell Accumulation and Glucose Intolerance. Cell reports.

[17] Audoy-Remus J (2008). Rod-Shaped monocytes patrol the brain vasculature and give rise to perivascular macrophages under the influence of proinflammatory cytokines and angiopoietin-2. J Neurosci.

[18] Leblond AL (2015). Systemic and Cardiac Depletion of M2 Macrophage through CSF-1R Signaling Inhibition Alters Cardiac Function Post Myocardial Infarction. PLoS One.

[19] Tsou CL (2007). Critical roles for CCR2 and MCP-3 in monocyte mobilization from bone marrow and recruitment to inflammatory sites. J Clin Invest.

[20] Franckhauser S (2002). Increased fatty acid re-esterification by PEPCK overexpression in adipose tissue leads to obesity without insulin resistance. Diabetes.

[21] Lin Y (2005). The hyperglycemia-induced inflammatory response in adipocytes: the role of reactive oxygen species. J Biol Chem.

[22] Obata A (2016). Tofogliflozin Improves Insulin Resistance in Skeletal Muscle and Accelerates Lipolysis in Adipose Tissue in Male Mice. Endocrinology.

[23] Nakano S (2015). Remogliflozin Etabonate Improves Fatty Liver Disease in Diet-Induced Obese Male Mice. J Clin Exp Hepatol.

[24] Qiang S (2015). Treatment with the SGLT2 inhibitor luseogliflozin improves nonalcoholic steatohepatitis in a rodent model with diabetes mellitus. Diabetol Metab Syndr.

[25] Ferrannini G (2015). Energy Balance After Sodium-Glucose Cotransporter 2 Inhibition. Diabetes Care.

[26] Roden M (2013). Empagliflozin monotherapy with sitagliptin as an active comparator in patients with type 2 diabetes: a randomised, double-blind, placebo-controlled, phase 3 trial. Lancet Diabetes Endocrinol.

[27] Bolinder J (2012). Effects of dapagliflozin on body weight, total fat mass, and regional adipose tissue distribution in patients with type 2 diabetes mellitus with inadequate glycemic control on metformin. J Clin Endocrinol Metab.

[28] Tosaki T (2017). Sodium-glucose Co-transporter 2 Inhibitors Reduce the Abdominal Visceral Fat Area and May Influence the Renal Function in Patients with Type 2 Diabetes. Intern Med.

[29] Zhuge F (2016). DPP-4 Inhibition by Linagliptin Attenuates Obesity-Related Inflammation and Insulin Resistance by Regulating M1/M2 Macrophage Polarization. Diabetes.

[30] Jing Yuanyuan, Wu Fan, Li Dai, Yang Lei, Li Qi, Li Rong (2018). Metformin improves obesity-associated inflammation by altering macrophages polarization. Molecular and Cellular Endocrinology.

[31] Parathath S (2011). Diabetes adversely affects macrophages during atherosclerotic plaque regression in mice. Diabetes.

[32] Satoh T (2013). Critical role of Trib1 in differentiation of tissue-resident M2-like macrophages. Nature.

[33] Nawaz A (2017). CD206(+) M2-like macrophages regulate systemic glucose metabolism by inhibiting proliferation of adipocyte progenitors. Nature communications.

[34] Sugita S, Kamei Y, Oka J, Suganami T, Ogawa Y (2007). Macrophage-colony stimulating factor in obese adipose tissue: studies with heterozygous op/+ mice. Obesity (Silver Spring).

[35] Steel JC, Waldmann TA, Morris JC (2012). Interleukin-15 biology and its therapeutic implications in cancer. Trends Pharmacol Sci.

[36] Barra NG (2010). Interleukin-15 contributes to the regulation of murine adipose tissue and human adipocytes. Obesity (Silver Spring).

[37] Shimazu T (2013). Suppression of oxidative stress by beta-hydroxybutyrate, an endogenous histone deacetylase inhibitor. Science.

[38] Youm YH (2015). The ketone metabolite beta-hydroxybutyrate blocks NLRP3 inflammasome-mediated inflammatory disease. Nat Med.

[39] Yang H, Tracey KJ (2005). High mobility group box 1 (HMGB1). Crit Care Med.

[40] Kimura T (2014). Responses of macrophages to the danger signals released from necrotic cells. Int Immunol.

[41] Rock KL, Lai JJ, Kono H (2011). Innate and adaptive immune responses to cell death. Immunol Rev.

[42] Tanaka M (2014). Macrophage-inducible C-type lectin underlies obesity-induced adipose tissue fibrosis. Nature communications.

[43] Olswang Y (2002). A mutation in the peroxisome proliferator-activated receptor gamma-binding site in the gene for the cytosolic form of phosphoenolpyruvate carboxykinase reduces adipose tissue size and fat content in mice. Proc Natl Acad Sci USA.

[44] Lee SY (2017). Adipocyte-Specific Deficiency of De Novo Sphingolipid Biosynthesis Leads to Lipodystrophy and Insulin Resistance. Diabetes.

