# Supplementary material for: A reduced M1-like/M2-like ratio of macrophages in healthy adipose tissue expansion during SGLT2 inhibition
Source: Sci Rep. 2018 Oct 31;8:16113. doi: 10.1038/s41598-018-34305-x (PMC6208395; doi:10.1038/s41598-018-34305-x)

SUPPLEMENTARY INFORMATION

A reduced M1-like/M2-like ratio of macrophages in healthy adipose tissue expansion during SGLT2 inhibition

Yasutaka Miyachi, Kyoichiro Tsuchiya, Kumiko Shiba, Kentaro Mori, Chikara Komiya, Naomi Ogasawara, and Yoshihiro Ogawa

SUPPLEMENTARY FIGURE LEGENDS

Supplementary Figure 1. Metabolic phonotypes in Ipra-treated WT mice fed a SD and a HFD, gating strategy and quantification of flow cytometry for ATMs, and gene expression analysis of peritoneal macrophages stimulated with conditioned medium of Epi.

(a) Blood glucose, serum insulin levels, body weight (BW), and weights of liver and epididymal adipose tissue (Epi) from Ipra-treated WT mice fed a HFD (n = 7–8). Blood samples were obtained from *ad libitum*-fed mice. (b) Blood glucose, serum insulin levels, BW, and weights of liver and Epi from Ipra-treated WT mice fed a standard diet (SD) (n = 6). (c) Stromal vascular fraction (SVF) of Epi was isolated after collagenase digestion. Cells were labelled with anti-CD45, anti-CD11b, and anti-F4/80 antibodies. Adipose tissue macrophages (ATMs) were defined as CD45+CD11b+F4/80+ cells in SVF. (d) Quantification of flow cytometry for total M1-like and M2-like ATMs normalized based on the weight of Epi from Ipra- or vehicle-treated HFD-fed mice (n = 4).(e) *Mrc1* and *Mgl2* mRNA levels in peritoneal macrophages (Mac) stimulated by adipose tissue conditioned medium (ATCM) from Ipra- or vehicle-treated HFD-fed mice (n = 4). All values are presented as mean ± SEM. *$p < 0.05$, ** p < 0.01, *** p < 0.001 vs. indicated group.

Supplementary Figure 2. Flow cytometric analysis of M1- and M2-like ATMs and M2-related gene expression in Epi of Ipra-treated WT mice fed a HFD with or without GW2580.

(a) Representative plots and quantification of flow cytometry for M1- and M2-like ATMs normalized based on the weight of Epi from Ipra-treated WT mice fed a HFD with or without GW2580 (n = 5, each group). (b) Gene expression levels in inguinal white adipose tissue (Inguinal) and brown adipose tissue (BAT) of Ipra-treated WT mice fed a HFD co-administered with vehicle (n = 5) or GW2580 (n = 5). (c) Correlation of Epi weight and the number of crown-like structures (CLS) (n = 10). All values are presented as mean ± SEM. *$p < 0.05$, ** $p < 0.01$ vs. Ipra + vehicle.

Supplementary Figure 3. Flow cytometric analysis of M1- and M2-like ATMs, surface CCR2 expression on monocytes, and liver enzyme levels in Ipra-treated mice fed a HFD with or without CCR2 KO background.

(a) Quantification of flow cytometry for total ATMs, and M1- and M2-like ATMs normalized based on the weight of Epi. (n = 5-6) (b) Representative plots and quantification of flow cytometry for M1- and M2-like ATMs of HFD-fed WT (n = 5) and CCR2 KO mice (n = 6) treated with Ipra. (c) Surface CCR2 expression on monocytes from a HFD-fed mouse treated with Ipra or vehicle. (d) Liver weight corrected for body weight (BW). (e) Serum alanine aminotransferase (ALT) levels. All values are presented as mean ± SEM. * $p < 0.05$ vs. WT.

Supplementary Figure 4. Full blots of phosphorylated Akt (pAkt) and Akt in Supplementary Figure 3h

Supplementary Figure 5. Gene expression in adipocytes, peritoneal macrophages, and Epi.

(a) *Il15* mRNA in isolated adipocytes from Epi of HFD-fed mice after 4 weeks of Ipra or vehicle treatment. (b) Representative images of IL-15 positive cells in Epi of HFD-fed WT mice after 4 weeks of Ipra or vehicle treatment. Scale bar = 100 μm. (c) *Il1b*, *Il6*, and *Tnfa* mRNA levels in the peritoneal macrophages stimulated by LPS (1 ng/ml) with or without pretreatment with beta-hydroxybutyrate (BHB, 1-10 mM) (n = 4). (d) *Il15* mRNA levels in the peritoneal macrophages (Mac) stimulated by vehicle, IL-4 (10 ng/ml), or IL-13 (10 ng/ml) (n = 4). (e) *Pck1* mRNA levels in Epi of HFD-fed WT (n = 5) and CCR2 KO (n = 6) mice treated by Ipra. (f) *Pck1* mRNA levels in Epi of SD or HFD-fed mice (n = 5). All values are presented as mean ± SEM. * $p < 0.05$, ** $p < 0.01$, *** $p < 0.001$ vs. indicated group.

Supplementary Figure 6. Ceramides and sphingomyelin subspecies in Epi of Ipra-treated WT and CCR2 KO mice fed a HFD, and WT mice fed a ketogenic diet.

Ceramide (CER) and sphingomyelin (SM) subspecies in Epi of (a) HFD-fed WT mice after 4 weeks of Ipra or vehicle treatment (n = 5), (b) Ipra-treated HFD-fed WT and CCR2 KO mice (n = 5), and (c) WT mice fed a ketogenic or control diet for 4 weeks (n = 5). All values are presented as mean ± SEM. * $p < 0.05$, ** $p < 0.01$, *** $p < 0.001$ vs. indicated group.

Supplementary Table 1. List of 44 genes belonging to the GO term “inflammatory response”

Official Gene symbol	Gene name	Fold change
Clec7a	C-type lectin domain family 7, member a	−1.65
Cd14	CD14 antigen	−1.42
Cd180	CD180 antigen	−1.54
Cd5l	CD5 antigen-like	−1.42
Nlrc4	NLR family, CARD domain containing 4	−1.62
Naip2	NLR family, apoptosis inhibitory protein 2	−1.4
Naip5	NLR family, apoptosis inhibitory protein 5	−1.52
Naip6	NLR family, apoptosis inhibitory protein 6	−1.5
Nlrp1b	NLR family, pyrin domain containing 1B	−1.5
Aim2	Absent in melanoma 2	−1.74
Aif1	Allograft inflammatory factor 1	−1.38
Camk1d	Calcium/calmodulin-dependent protein kinase ID	−1.43
Ccl3	Chemokine (C-C motif) ligand 3	−1.63
Ccr3	Chemokine (C-C motif) receptor 3	−2.94
Ccr5	Chemokine (C-C motif) receptor 5	−1.61
Csf1r	Colony stimulating factor 1 receptor	−1.41
C3ar1	Complement component 3a receptor 1	−1.49
C5ar1	Complement component 5a receptor 1	−1.62
Cybb	Cytochrome b-245, beta polypeptide	−1.57
Fpr-rs3	Formyl peptide receptor, related sequence 3	−1.45
Havcr2	Hepatitis A virus cellular receptor 2	−1.66
Hmgb2	High mobility group box 2	−1.38
Il1rap	Interleukin 1 receptor accessory protein	−1.33
Il15	Interleukin 15	−1.57
Kit	Kit oncogene	−1.38
Ly86	Lymphocyte antigen 86	−1.62
Ncf1	Neutrophil cytosolic factor 1	−1.55
Olr1	Oxidized low density lipoprotein (lectin-like) Receptor 1	−1.47
Pik3cd	Phosphatidylinositol 3-kinase catalytic delta Polypeptide	−1.47

Pik3cg	Phosphoinositide-3-kinase, catalytic, gamma Polypeptide	−1.48
Pla2g7	Phospholipase A2, group VII (platelet-activating Factor acetylhydrolase, plasma)	−1.44
P2rx7	Purinergic receptor P2X, ligand-gated ion channel, 7	−1.45
Spp1	Secreted phosphoprotein 1	−1.91
Stab1	Stabilin 1	
Tlr1	Toll-like receptor 1	−1.86
Tlr11	Toll-like receptor 11	−1.67
Tlr13	Toll-like receptor 13	−1.95
Tlr7	Toll-like receptor 7	−1.95
Tlr8	Toll-like receptor 8	−1.44
Ticam2	Toll-like receptor adaptor molecule 2	−1.88
Tpsb2	Tryptase beta 2	−2.81
Tnfrsf1b	Tumor necrosis factor receptor superfamily, member 1b	−1.44
Tnf	Tumor necrosis factor	−1.4

Negative fold change shows a downregulation of indicated genes by ipragliflozin administration.

SUPPLEMENTARY MATERIAL AND METHODS

Reagents

All reagents were purchased from Sigma-Aldrich (St. Louis, MO, USA) or Nacalai Tesque (Kyoto, Japan), unless otherwise noted.

Preparation of adipose tissue conditioned medium and cell culture

Epididymal fat pads from HFD-fed mice after 4 weeks of Ipra or vehicle treatement were incubated at DMEM containing 0.25% BSA and 1% penicillin/streptomycin (0.25% BSA/DMEM) for 20 h. Conditioned medium was collected and diluted two fold with 0.25% BSA/DMEM. Thioglycollate-elicited peritoneal macrophages were cultured in 12-well plates and incubated for 24 h with 0.25% BSA/DMEM (control) or the diluted conditioned medium.

Lipidomics analysis

Lipids were extracted from samples using dichloromethane and methanol in a modified Bligh-Dyer extraction in the presence of internal standards with the lower, organic, phase being used for analysis. The extracts were concentrated under nitrogen and reconstituted in 0.25mL of dichloromethane:methanol (50:50) containing 10mM ammonium acetate. The extracts were placed in vials for infusion-MS analyses, performed on a SelexION equipped Sciex 5500 QTRAP using both positive and negative mode electrospray. Each sample was subjected to 2 analyses, with IMS-MS conditions optimized for lipid classes monitored in each analysis. The 5500 QTRAP was operated in MRM mode to monitor the transitions for over 1,100 lipids. Individual lipid species were

quantified based on the ratio of signal intensity for target compounds to the signal intensity for an assigned internal standard of known concentration. Fatty acid compositions were determined by calculating the proportion of individual fatty acids within each class.

Supplementary Figure 1

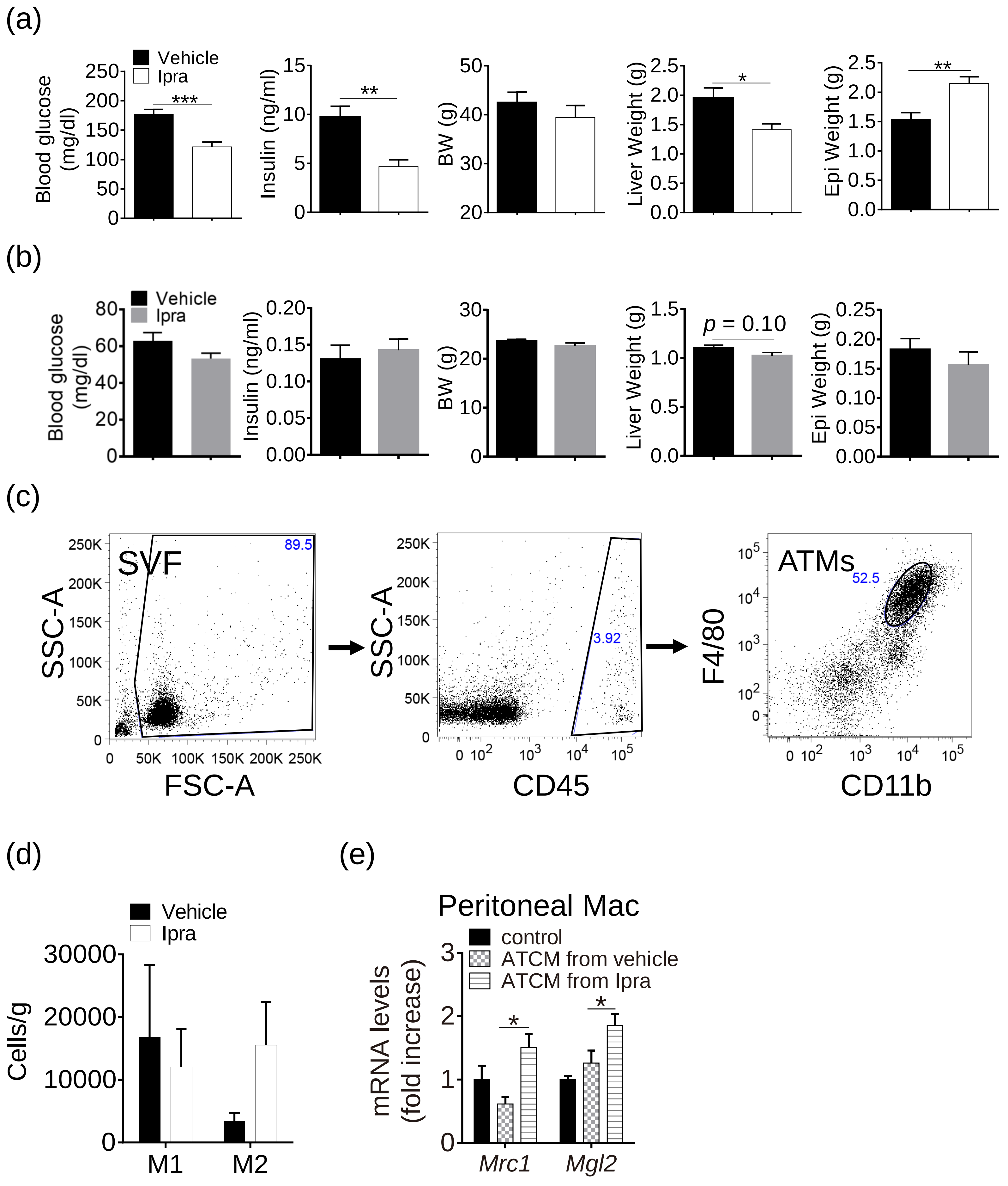

Supplementary Figure 2

(a)

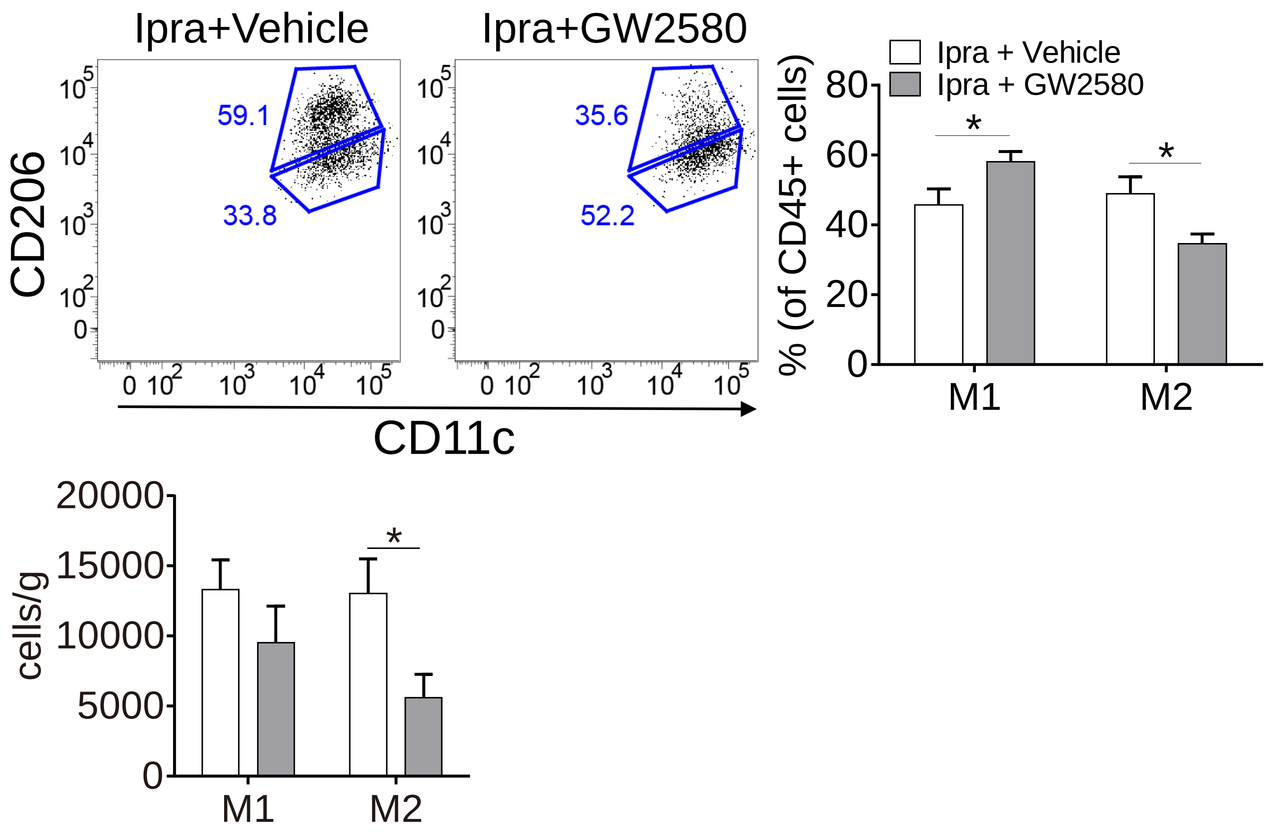

(b)

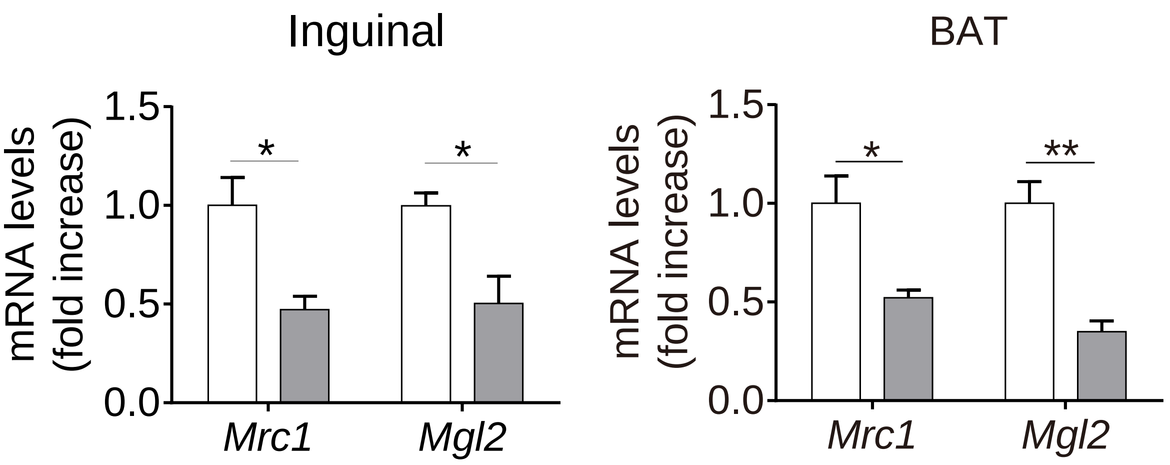

(c)

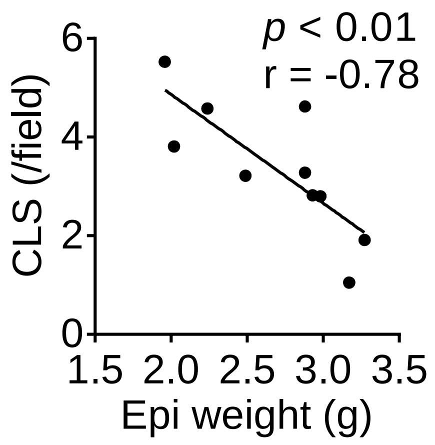

Supplementary Figure 3

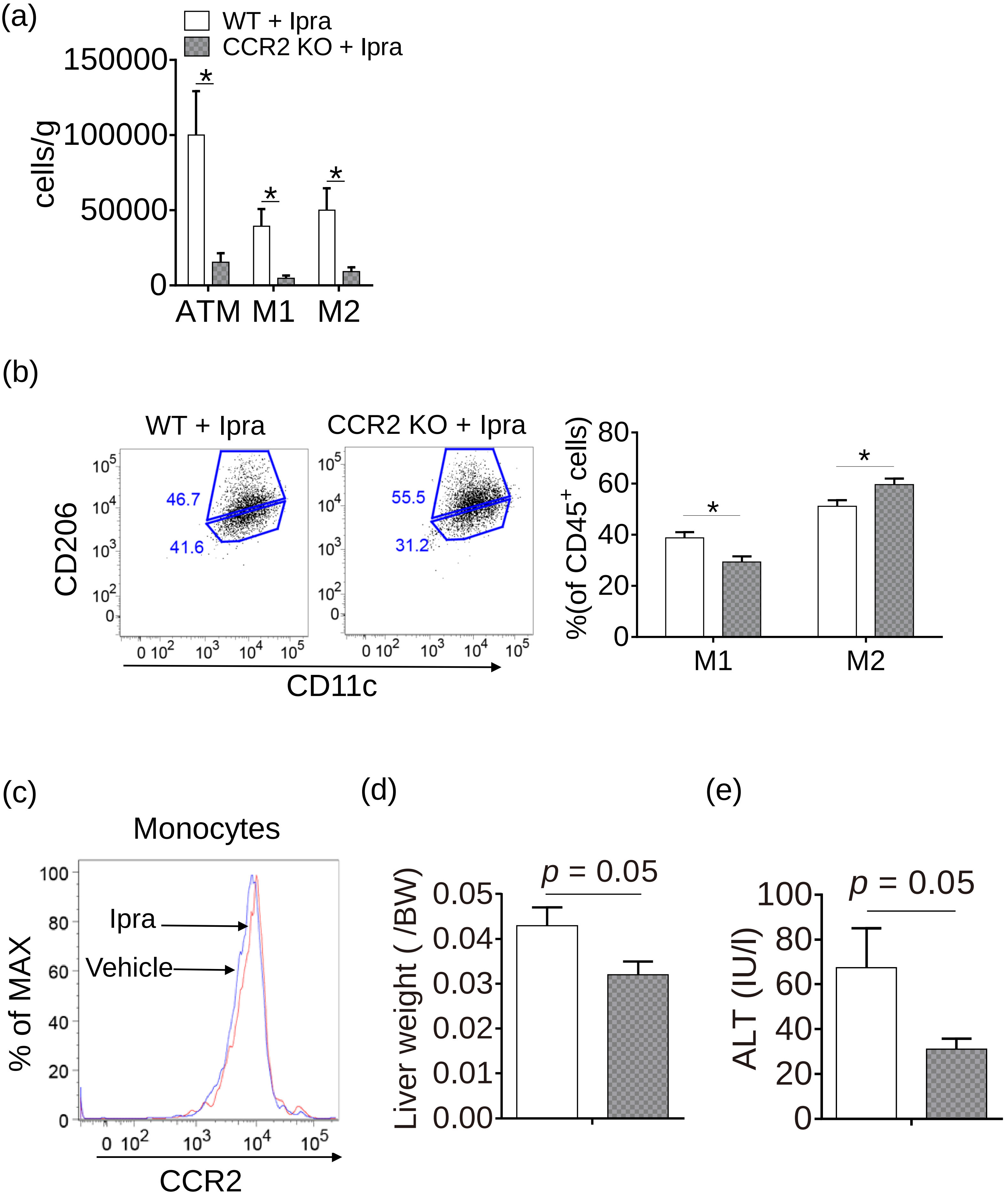

Supplementary Figure 4

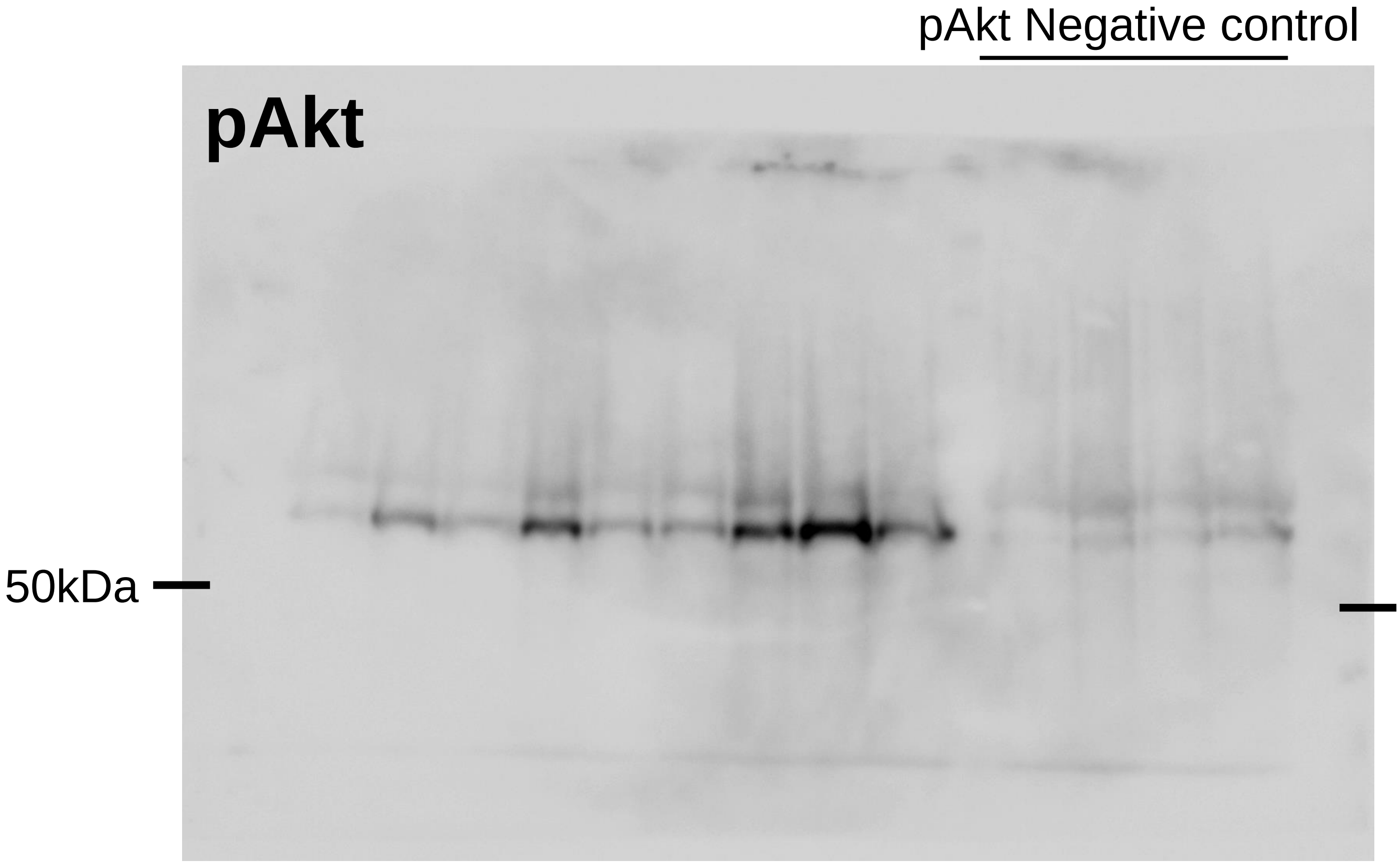

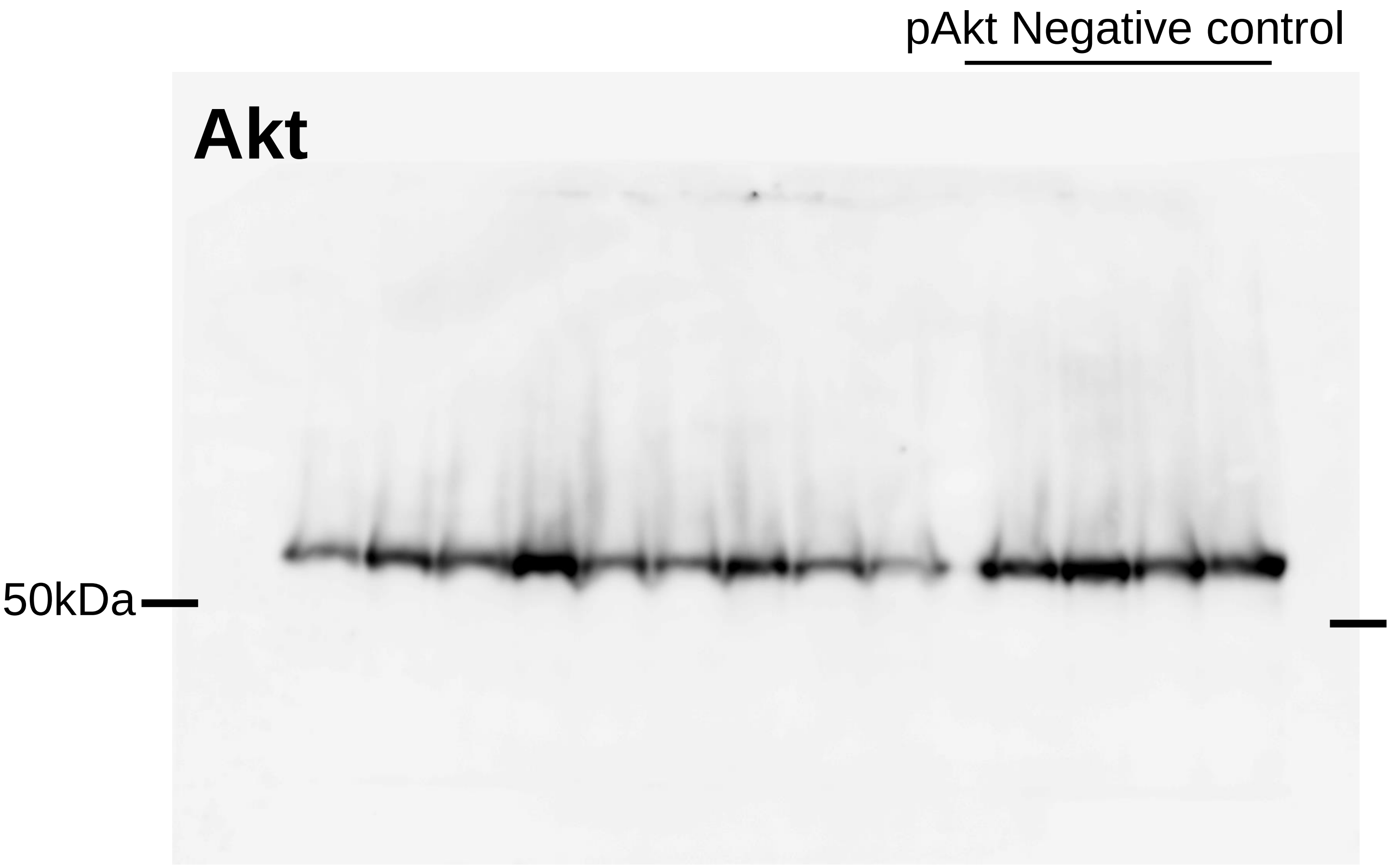

Supplementary Figure 5

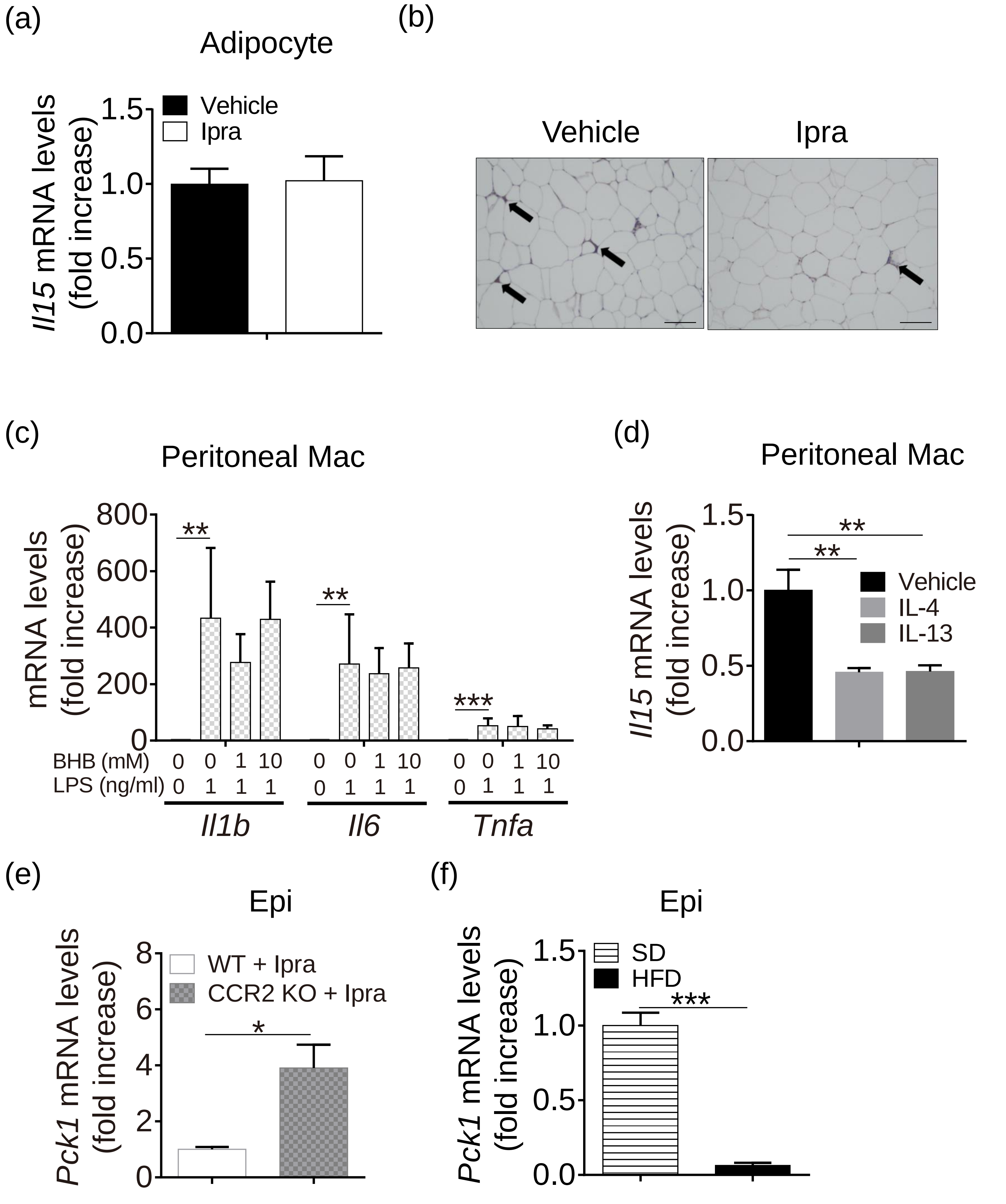

Supplementary Figure 6

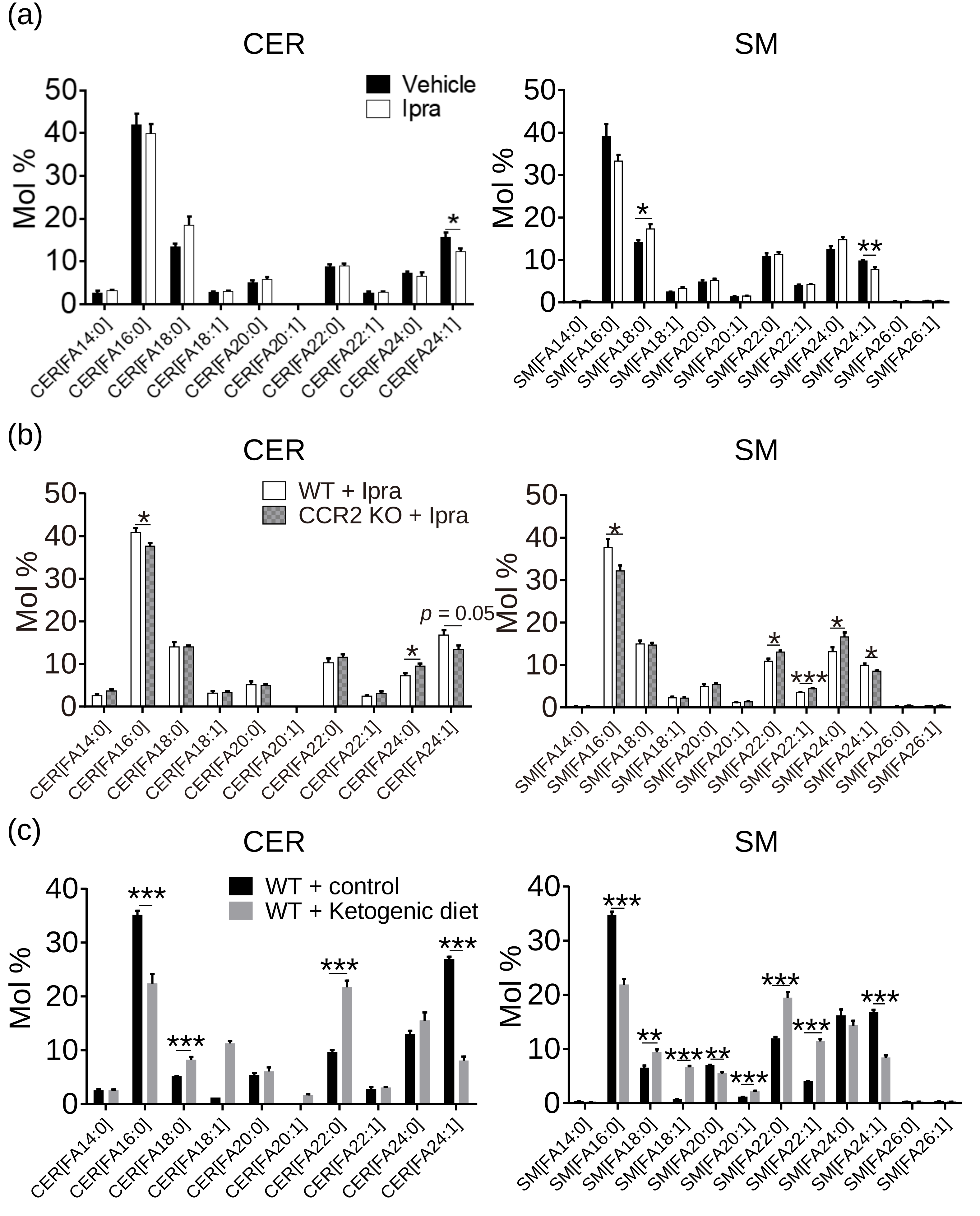

Supplement: Supplementary file 1 — Supplementary Information [file 41598_2018_34305_MOESM1_ESM.pdf]
